# Strategies for Success. Viral Infections and Membraneless Organelles

**DOI:** 10.3389/fcimb.2019.00336

**Published:** 2019-10-11

**Authors:** Aracelly Gaete-Argel, Chantal L. Márquez, Gonzalo P. Barriga, Ricardo Soto-Rifo, Fernando Valiente-Echeverría

**Affiliations:** ^1^Molecular and Cellular Virology Laboratory, Virology Program, Institute of Biomedical Sciences, Faculty of Medicine, Universidad de Chile, Santiago, Chile; ^2^HIV/AIDS Workgroup, Faculty of Medicine, Universidad de Chile, Santiago, Chile; ^3^Emerging Viruses Laboratory, Virology Program, Institute of Biomedical Sciences, Faculty of Medicine, Universidad de Chile, Santiago, Chile

**Keywords:** RNAstasis, RNA granules, membraneless organelles, stress granules, P-Bodies, anti-viral host immune response

## Abstract

Regulation of RNA homeostasis or “RNAstasis” is a central step in eukaryotic gene expression. From transcription to decay, cellular messenger RNAs (mRNAs) associate with specific proteins in order to regulate their entire cycle, including mRNA localization, translation and degradation, among others. The best characterized of such RNA-protein complexes, today named membraneless organelles, are Stress Granules (SGs) and Processing Bodies (PBs) which are involved in RNA storage and RNA decay/storage, respectively. Given that SGs and PBs are generally associated with repression of gene expression, viruses have evolved different mechanisms to counteract their assembly or to use them in their favor to successfully replicate within the host environment. In this review we summarize the current knowledge about the viral regulation of SGs and PBs, which could be a potential novel target for the development of broad-spectrum antiviral therapies.

## Introduction

RNA plays key roles in all biological systems where RNAstasis is a *central processing unit* in the regulation of gene expression in eukaryotic cells (Sharp, 2009). RNAstasis include synthesis, modification, protection, storage, release, transportation and degradation of different types of RNA (mRNA, tRNA, rRNA, siRNA, miRNA, lncRNA, piRNA, snRNA, snoRNA, smRNA) and metabolic processes mediated by RNA–protein complexes called RNA granules. Depending on its localization, RNA granules are found in the nucleus, in the nucleolus, paraspeckles, nuclear speckles and Cajal bodies; or in the cytoplasm, as stress granules (SGs) and processing bodies (PBs). All are membraneless organelles (i.e., lack an enclosing membrane, MLOs) to allow for rapid exchange of components with the surrounding cellular environment (Fay and Anderson, 2018). MLOs contain a heterogeneous mixture of nucleic acids and proteins that present low-complexity regions (LCRs) and intrinsically disordered regions (IDRs) regulated by post-translational modifications (Ramaswami et al., 2013; Panas et al., 2016; Wheeler et al., 2016). MLO biogenesis has been shown to be via liquid–liquid phase separation (LLPS) process, supporting the high flexibility and quick adaptive responses to environmental stresses required for function (reviewed in Fay and Anderson, 2018).

After several rounds of translation, an mRNA undergoes degradation as a way of turnover. Indeed, it is suggested that mRNA degradation is tightly dependent on translation (Bicknell and Ricci, 2017).

However, under conditions of cellular stress, the cell responds by mounting a robust response causing the shutoff of protein synthesis in order to protect the mRNA so that translation can resume once the stress disappears. Repression of gene expression induces the assembly of RNA granules such as SGs and PBs, which are involved in mRNA triage and untranslated mRNA storage, respectively. By using single mRNA imaging in living human cells, it has been recently reported that a single mRNA can interact with both SGs and PBs (Wilbertz et al., 2018; Moon et al., 2019). However, while Wilbertz et al. showed that an mRNA preferably moves from a SG to a PB, Moon et al. showed a dynamic and bidirectional exchange of a single mRNA to multiple SGs and PBs (Wilbertz et al., 2018; Moon et al., 2019). Despite their distinctive organization and unique molecular markers, SGs and PBs share molecular components which could allow the dynamic shuttling of an mRNA between them (Kedersha et al., 2005).

**Figure 1:**
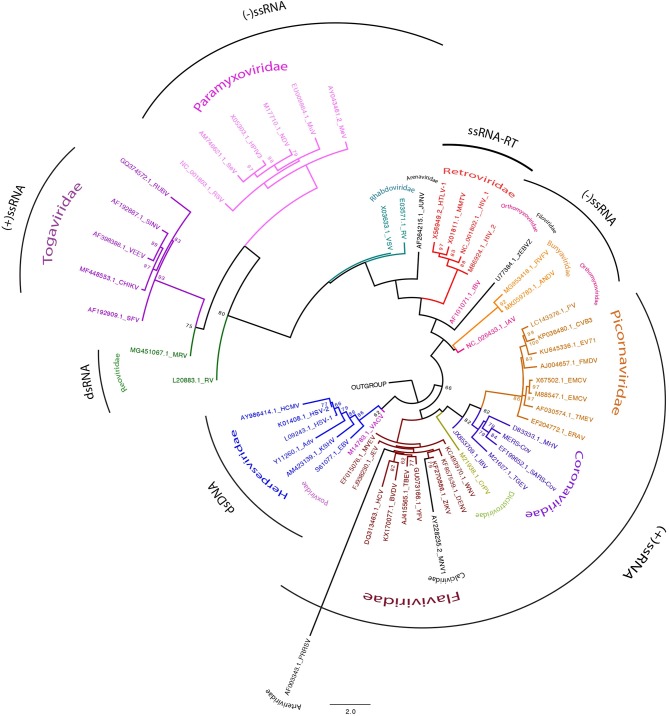
Viral families tree. Phylogenetic tree showing 56 sequences representing all viral families described to modulate RNA granules assembly. The chosen sequences were “gene encoding to superficies structural protein.” The sequences were selected from NCBI databases (https://www.ncbi.nlm.nih.gov/nuccore/). Alignment were performed by MUSCLE (http://www.drive5.com/muscle/) (Edgar, 2004). Phylogenetic tree was constructed with MEGA6 (http://www.megasoftware.net) and IQ-TREE on the IQ-TREE web server (http://www.cibiv.at/software/iqtree/) (Trifinopoulos et al., 2016) by using the maximum-likelihood (ML) method. Robustness of tree topologies was assessed with 1,000 bootstrap replicates. Phylogenetic tree was constructed using ML inference with the general time reversible (GTR)_G nucleotide substitution model. Viral families are showed in different colors. Genomes by clade are grouped by black arch.

Viral infections are a major trigger of cellular stress and, thus, viruses have evolved diverse mechanisms aimed to modulate host RNAstasis with a direct impact in the assembly of different RNA granules while counteracting mRNA decay machineries in order to ensure viral replication (Poblete-Durán et al., 2016; Toro-Ascuy et al., 2016). In this review, we provide an update on the current knowledge of the different strategies used by several virus families to modulate the RNA granules assembly/disassembly, specifically SGs and PBs, in order to promote a successful viral infection (see Figure 1).

## Viral Families and Stress Granules

SGs are translationally silent membraneless organelles with a diameter between 0.1 and 4 μm. Canonical or *bona fide* SGs contain mRNA, RNA-binding proteins, and components of the 40S ribosomal subunit. Many proteins involved in SG assembly are RNA binding proteins that favor mRNA stability (TIA-1, TIAR, HuR), mRNA metabolism (G3BP-1, G3BP-2, DDX6, SMN, Staufen1, DHX36, Caprin1, ZBP1, HDAC6, ADAR), signaling proteins (mTOR, RACK1) and interferon-stimulated gene (ISG) products (PKR, ADAR1, RIG-I, RNase L, and OAS (reviewed in Poblete-Durán et al., 2016). Recently, Nunes et al. generated an open access electronic resource containing all SGs-recruited protein reported to date (available at https://msgp.pt/) (Nunes et al., 2019). Its assembly is typically a consequence of translation repression upon phosphorylation of the translation initiation factor eIF2α by environmental stress such as heat shock, UV irradiation, oxidative stress, viral infection, and even upon treatment with several drugs (see Table 1). Most of these stresses are sensed by the eIF2α kinases PKR, which is activated by double-stranded RNA during viral infection (Williams, 2001); PERK, which is activated upon accumulation of misfolded protein in the ER and during hypoxia (Harding et al., 2000); HRI, which is activated by oxidative stress and heme deprivation (Han et al., 2001); and GCN2, which is activated by aminoacid deprivation and UV irradiation (Jiang and Wek, 2005). However, SGs can also be formed by inhibitors of translation that target other components of the translation machinery (Table 1) or by overexpression of SG-associated proteins such as TIA-1/TIAR or G3BP-1 (Kedersha et al., 1999; Tourrière et al., 2003). In addition to its role in mRNA triage, SGs have been described as signaling centers. Recruitment of signaling proteins to SGs allow the crosstalk between multiple stress cascades including translational control pathways, prevention of apoptosis and innate immune responses against viral infections (reviewed in Kedersha et al., 2011; Onomoto et al., 2014; Mahboubi and Stochaj, 2017).

**Table 1:** List of drugs/stressors used to induce or disassemble SGs and PBs.

**Class**	**Drug/stressor**	**Effect**	**Mechanism**	**References**
	Pateamine-A	Induces SG assembly	Interacts with eIF4A disrupting the eIF4F complex	Bordeleau et al., 2005; Kedersha et al., 2006
	Hippuristanol	Induces SG assembly	Inhibits eIF4A RNA binding activity	Bordeleau et al., 2006; Mazroui et al., 2006
Translation inhibitors	Cycloheximide	Disassembles both SGs and PBs	Inhibits eEF2-mediated translation elongation	Obrig et al., 1971; Mollet et al., 2008
	Selenite	Induces non-canonical SG assembly	Enhances 4EBP-1 binding to eIF4E, thus disrupting the eIF4F complex	Fujimura et al., 2012
	Sorbitol	Induces SG assembly	Causes osmotic stress, which enhances 4EBP-1 binding to eIF4E, thus disrupting the eIF4F complex	Patel et al., 2002
	Arsenite	Induces SG and PB assembly	Induces HRI-mediated eIF2α phosphorylation^*^	McEwen et al., 2005
	Dithiothreitol (DTT)	Induces SG assembly	Induces PERK-mediated eIF2α phosphorylation	Oslowski and Urano, 2011; Dimasi et al., 2017
	Heat-Shock	Induces SG assembly and inhibits PBs	Induces HRI-mediated eIF2α phosphorylation	McEwen et al., 2005; Aulas et al., 2017
eIF2α kinases Stressors	Poly I:C	Induces SG assembly	Induces PKR-mediated eIF2α phosphorylation	Weissbach and Scadden, 2012
	Bortezomib and MG132 (proteosome inhibitors)	Induce SG assembly	Induce HRI(Bortezomib)- and GCN2(MG132)- mediated eIF2α phosphorylation	Mazroui et al., 2007; Fournier et al., 2010
	Thapsigargin	Induces SG assembly	Induces PERK-mediated eIF2α phosphorylation	Kimball et al., 2002
eIF2α modulators	ISRIB	Inhibit SG assembly	Prevents eIF2B inhibition, maintaining translation initiation despite eIF2α phosphorylation	Sidrauski et al., 2015
	Salubrinal	Induces SG assembly	Blocks eIF2α dephosphorylation	Boyce et al., 2005
Others	1,6-Hexanediol	Disassembles and induces PB and SG assembly	Disrupt weak hydrophobic interactions causing quick disassembly of granules that reappear after a few minutes^**^	Wheeler et al., 2016; Kroschwald et al., 2017
	Zn^+2^	Stress- inducible second messenger	Induces reversible multimerization, phase separation and SG recruitment of TIA-1	Rayman et al., 2018

* *Arsenite can also induce SG assembly independent of HRI. In Drosophila, which lacks HRI, induces the PEK eIF2a kinase (Farny et al., 2009). In addition, Sharma et al. demonstrated that arsenite can also induce phosphorylation of PKR and SG assembly in HeLa and BCBL-1 cells (Sharma et al., 2017)*.

** *It has been shown to also alter many other cellular structures (Wheeler et al., 2016)*.

Here, we summarize how viruses modulate SG accumulation in order to maintain viral protein synthesis and particles production.

### Double-Stranded DNA (dsDNA) Viruses

#### Herpesviridae

All members of the *Herpesviridae* family that have been studied prevent the accumulation of SGs. Herpes simplex virus type 1 (HSV-1) infection upregulates and relocalizes to the cytoplasm the SG components TTP, TIAR, and TIA-1 but does not induce SG assembly (Esclatine et al., 2004). The virion host shutoff (vhs) protein, an mRNA endonuclease, has been shown to be essential in SGs blockade as vhs-deficient HSV-1 (Δvhs) infected cells do trigger SG assembly (Esclatine et al., 2004; Dauber et al., 2011, 2016). HSV vhs is thought to facilitate viral mRNA translation throughout the viral cycle by reducing host mRNAs and preventing viral mRNA overload (Dauber et al., 2016). Δvhs-induced SGs accumulation correlates with increased PKR activation (Sciortino et al., 2013; Dauber et al., 2016; Burgess and Mohr, 2018), but while a group observed higher eIF2α phosphorylation (Pasieka et al., 2008; Burgess and Mohr, 2018), others did not (Dauber et al., 2011, 2016). This phenotype could be in part due the reduced levels of the late-expressed dsRNA binding protein Us11, that has been shown to block PKR activation (Mulvey et al., 2003; Dauber et al., 2011). Burgess and Mohr showed that dsRNA accumulates and partially localizes to Δvhs-induced SGs (Burgess and Mohr, 2018). Furthermore, they show that SGs are not assembled neither PKR is phosphorylated in Δvhs-infected cells upon treatment with ISRIB (see Table 1) or in absence of G3BP-1 or TIA-1. Based on these observations, the authors suggest that Δvhs-enhanced PKR activation is a consequence of SG assembly due to dsRNA accumulation (Burgess and Mohr, 2018). Interestingly, other HSV proteins have also been involved in SGs regulation, although it is not clear whether they all act cooperatively, or during different stages of the viral cycle. HSV-1 ICP27 has been shown to prevent formation of arsenite-induced SGs by inhibiting PKR and eIF2α phosphorylation (Sharma et al., 2017). On the other hand, overexpression of HSV-1 ICP8 protein, a G3BP binding partner, blocks arsenite-induced SG assembly (Panas et al., 2015). Similar to HSV-1, SGs do not assemble during herpes simplex virus 2 (HSV-2) infection and its blockade is mediated by the vhs protein (Finnen et al., 2012; Dauber et al., 2014). HSV-2 vhs protein has also been shown to shutoff protein synthesis by depleting mRNAs (Smith et al., 2000). Wild Type (WT) HSV-2 impairs arsenite-induced SGs despite increased eIF2α phosphorylation, but not pateamine (eIF2α-independent)-induced SGs, indicating that vhs can disrupt or modify SGs independently of eIF2α phosphorylation (Finnen et al., 2012). Further investigation revealed that (i) vhs localizes to SGs, (ii) vhs not only inhibits SG assembly but also disrupts pre-assembled SGs, and (iii) vhs endoribonuclease activity is required in SGs modulation (Finnen et al., 2016). Interestingly, TIA-1 was shown to egress before G3BP in the course of vhs-mediated SGs disassembly, which could be explained by the G3BP-enriched core SG structure (Jain et al., 2016; Niewidok et al., 2018). Based on these results, the authors proposed that HSV-2 vhs modify SGs by directly or indirectly degrading mRNA. Human cytomegalovirus (HCMV) inhibits the assembly of SGs but induces the unfolded protein response (UPR) (Isler et al., 2005a). Typically, this ER stress response leads to eIF2α phosphorylation via PERK activation, but HCMV limits eIF2α phosphorylation without diminishing PERK activation (Isler et al., 2005b). Marshall et al. showed that infection with HCMV lacking pTRS1 and pIRS1, dsRNA-binding proteins linked to PKR pathway inhibition, results in increased levels of eIF2α phosphorylation and the reduction of viral protein synthesis (Marshall et al., 2009). Both proteins have identical amino-terminal regions and share 35% of similarity in their carboxy-terminal regions, suggesting that HCMV pTRS1 and pIRS1 have redundant roles in evading dsRNA-mediated antiviral response. Ziehr et al. demonstrated that lack of both proteins also results in PKR activation and SG assembly, and that expression of either pTRS1 or pIRS1 is necessary and sufficient to prevent PKR activation, eIF2α phosphorylation and SG assembly (Ziehr et al., 2016). Furthermore, pTRS1 PKR binding domain (PDB) was shown to be critical to accomplish those three phenotypes suggesting that the main mechanism of HCMV to inhibit SG assembly is through PKR antagonism. Strikingly, pTRS1 transfection interferes with arsenite-induced SG assembly in WT and PKR-depleted cells, but pTRS1-ΔPBD does not, suggesting that pTRS1 could also obstruct SG assembly promoted by other eIF2α kinases and that its PDB is crucial for it. Kaposi's sarcoma-associated herpesvirus (KSHV) does not lead to SG accumulation via the viral protein ORF57 and SOX which are able to restrict arsenite-induced SG assembly independently (Sharma et al., 2017). ORF57, the HSV-1 ICP27 homologous, inhibits PKR/eIF2α phosphorylation by directly interacting with PKR via its N-terminal dsRNA-binding domain, and with PACT via its two N-terminal RNA-binding motifs, thus obstructing PKR binding to both dsRNA and PACT. The mechanism and the spatiotemporal regulation of SG assembly by the viral shutoff exonuclease SOX is unclear, but it might be related to its intrinsic RNA endonuclease activity similarly to HSV-2 vhs (Glaunsinger and Ganem, 2004; Sharma et al., 2017). Expression of Epstein–Barr virus (EBV) protein EB2, the counterpart of KSHV ORF57 and HSV-1 ICP27, does not abolish SG assembly, neither PKR/eIF2α phosphorylation, indicating that this specific ability to regulate SGs is not conserved along herpesviruses (Sharma et al., 2017). Further research is necessary to define the effect of EBV on SG assembly.

#### Poxviridae

Unlike herpes viruses, Vaccinia virus (VACV), a member of *Poxviridae* family, exploits SG components to favor viral protein production (reviewed in Liem and Liu, 2016). VACV redistributes proteins from the host translation machinery and SGs, such as eIF4E, eIF4G, G3BP, and Caprin1 into viral replication factories (RFs) assembled in the cytoplasm of the host cell (Katsafanas and Moss, 2004, 2007). Notably, TIA-1 is not recruited into these replication foci (Walsh et al., 2008). How VACV redistributes each of these components remain unclear, but evidences have shown that VACV ssDNA-binding protein I3 associates and recruits eIF4G to ssDNA formed within the RFs (Zaborowska et al., 2012). Furthermore, it was shown that G3BP-1 and Caprin1 associate with nascent VACV DNA by mass spectrometry (Senkevich et al., 2017). Despite the disruption of canonical SGs for its own benefit, infection with the replication-defective VACV lacking E3L leads to the accumulation of granule-like structures around the RFs, named antiviral granules (AVGs), that arrest viral translation (Simpson-Holley et al., 2010). AVGs contain proteins that are typically found in SGs such as TIA-1, eIF3b, G3BP-1, and USP10, but they are not affected by cycloheximide, a drug that induce the disassembly of *bona fide* SGs (Simpson-Holley et al., 2010). AVG assembly requires eIF2α phosphorylation via PKR activation (Simpson-Holley et al., 2010; Pham et al., 2016), process that is inhibited in presence of E3L (Chang et al., 1992). Furthermore, TIA-1 is an essential component of AVGs, as in its absence these antiviral granules are not formed even if PKR and eIF2α are phosphorylated (Simpson-Holley et al., 2010). Interestingly, WT VACV infection also induces AVGs assembly that repress viral protein synthesis but to negligible levels (Rozelle et al., 2014). Recently, another mutant VACV lacking C7L/K1L was shown to induce AVG assembly (Liu and McFadden, 2014). AVGs accumulation was abolished and abortive infection was rescued in ΔC7L/K1L in SAMD9-depleted cells, suggesting that C7L/K1L antagonize SAMD9 host protein antiviral function. Even though SAMD9 localize to both ΔE3L and ΔC7L/K1L VACV induced-AVGs, infectivity neither AVG assembly is blocked with ΔE3L VACV in SAMD9-depleted cells, suggesting a different mode of organization of the granules induced by both mutants (Liu and McFadden, 2014). ΔC7L/K1L-dependent AVGs assembly is independent of eIF2α phosphorylation, in contrast to ΔE3L AVG accumulation (Liu and McFadden, 2014). Viral mRNA was shown to colocalize with AVGs during ΔC7L/K1L VACV infection, thus limiting translation of viral proteins (Sivan et al., 2018), as well as dsRNA, TIA-1 and the viral protein E3L (Meng and Xiang, 2019). Despite of that, TIA-1 is not required for ΔC7L/K1L-mediated AVG assembly as it is on ΔE3L (Meng and Xiang, 2019). The role of each viral system, E3L and C7L/K1L, to prevent formation of AVGs in the context of viral infection remain to be studied.

### Double-Stranded RNA (dsRNA) Viruses

#### Reoviridae

Rotavirus replication, the prototypical member of the *Reoviridae* family, also occurs in viral replication factories and upon infection, synthesis of cellular proteins is reduced while viral protein production is maintained. Accumulation of viral dsRNA in the cytoplasm causes a persistent PKR-dependent eIF2α phosphorylation, even when eIF2α phosphorylation is not required for viral replication (Montero et al., 2008; Rojas et al., 2010). Despite that, rotavirus infection does not induce SG assembly; instead it changes the cellular localization of SG components (Montero et al., 2008). TIA-1 is relocalized to the cytoplasm, eIF4E distributes more homogeneously in the cytoplasm, and PABP is translocated to the nucleus through the viral protein NSP3 (Montero et al., 2008). Recently, Dhillon et al. determined that rotavirus remodels SGs by excluding some of their proteins, such as G3BP-1, hnRNP A1, and ZBP1, and then recruits these atypical granules to viral replication factories (Dhillon and Rao, 2018; Dhillon et al., 2018). It will be of interest to understand how rotavirus selectively excludes these specific SG components. In contrast, uncoating of the mammalian orthoreovirus (MRV) during the early stage of infection leads to eIF2α phosphorylation by the action of at least two eIF2 kinases, suggesting that MRV infection is a complex process that induces different types of stresses to the cell (Qin et al., 2009). Phosphorylation of eIF2α triggers SG accumulation (Qin et al., 2009). MRV cores are then recruited into the assembled SGs, a step that depends on synthesis of viral mRNA. As the infection proceeds, assembled SGs are disrupted in order to allow efficient synthesis of viral proteins, despite the sustained levels of eIF2α phosphorylation (Qin et al., 2011). Like rotavirus, MRV replication occurs in viral replication factories that grow in the perinuclear region (Rhim et al., 1962). The non-structural μNS viral protein associates with σNS, σ2, μ2, and λ1 are recruited into viral replication factories. μNS viral protein has been shown to localize with SGs, although is unable to independently prevent SGs accumulation (Carroll et al., 2014). Interestingly, the SG components G3BP-1, Caprin1, USP10, TIAR, TIA-1, and eIF3b were found to localize to the outer peripheries of viral replication factories (Choudhury et al., 2017). σNS and μNS were shown to be responsible for their redistribution as well as for disruption of the SG assembly. In addition, in the absence of G3BP-1 the other recruited SGs-associated protein, except for eIF3b, do not localize to RFs. The recruitment mode is thought to be as follows: Caprin1, USP10, TIAR, and TIA-1 interact with G3BP-1 which binds to σNS via its RNA recognition (RRM) and an arginine/glycine-rich (RGG) motifs. Then, σNS partner with μNS for RF localization, carrying all the other proteins with it (Choudhury et al., 2017).

### Positive-Sense Single Stranded RNA ((+) ssRNA) Viruses

#### Picornaviridae

Members of the *Picornaviridae* family also modulate SGs accumulation during replication. Poliovirus (PV) regulates SGs in a time-dependent manner; at early times the 2A proteinase induces assembly of SGs (Mazroui et al., 2006; Chen et al., 2008) that are later disassembled by the 3C proteinase through G3BP-1 cleavage (White et al., 2007). Despite of *bona fide* SG disruption, atypical SGs (aSGs) that contain TIA-1 and viral RNA, but no eIF4G nor PABP, still accumulate later in the course of PV infection (Piotrowska et al., 2010; White and Lloyd, 2011). A similar temporal control of SG assembly is exhibited by Coxsakievirus B3 (CVB3) and Enterovirus 71 (EV71). CVB3 2A proteinase induces SG assembly as early as 3 h post infection (hpi) in an eIF2α phosphorylation-independent manner (Wu et al., 2014; Zhai et al., 2018). It has been described that they have an antiviral role, inhibiting the biosynthesis of CVB3 (Zhai et al., 2018). However, at 6 hpi CVB3 induces the assembly of granules that do not contain G3BP-1 or eIF4G, likely because of G3BP-1 cleavage (Fung et al., 2013; Zhai et al., 2018). In the case of EV71, canonical SGs are assembled early during infection dependent on the PKR-eIF2α pathway (Zhu et al., 2016), but are dispersed at late stages of infection (Yang et al., 2018b; Zhang et al., 2018). Yet, atypical SGs in which TIA-1, TIAR, Sam68, and viral RNA are persistently aggregated in an eIF2α independent and cycloheximide-resistant manner remain during infection. Yang et al. showed that EV71 2A protease expression is enough for atypical SGs induction through the cleavage of eIF4GI and *bona fide* SGs blockage by abolishing eF4GI-G3BP-1 interaction (Yang et al., 2018a,b). In contrast, Zhang et al. reported that EV71 3C protease alone is sufficient to inhibit canonical SGs accumulation during late stages of infection through G3BP-1 cleavage at amino acid Q326 (Zhang et al., 2018). Interestingly, cells infected with EV71-2AC110S (a cleavage-deficient 2A protease) do form canonical SGs in which viral RNA is aggregated, suggesting that EV71 blocks *bona fide* SGs but induce atypical SGs to facilitate viral translation by stalling only cellular mRNAs (Yang et al., 2018b; Zhang et al., 2018). Unlike already mentioned picornaviruses, encephalomyocarditis virus (EMCV) infection does not induce SG assembly at all, and cleavage of G3BP-1 is the mechanism for their disruption (Ng et al., 2013). On the other hand, the leader (L) protein of Theiler murine encephalomyelitis virus (TMEV) and mengovirus (a strain of EMCV) inhibit SG assembly without cleaving G3BP-1 (Borghese and Michiels, 2011; Langereis et al., 2013). A mutant mengovirus, in which the Zn-finger domain of L is disrupted, induces antiviral G3BP-1 aggregations in which Caprin-1 and PKR are recruited, resulting in PKR activation and viral replication inhibition (Langereis et al., 2013; Reineke et al., 2015). Foot and Mouth Disease Virus (FMDV) does not induce SG assembly despite strongly shutoff cap-dependent translation and G3BP-1 dephosphorylation at Ser-149 (Ye et al., 2018; Visser et al., 2019), suggesting that FMDV infection regulates the cellular stress response. In fact, G3BP-1, eIF4G, eIF3, and eIF2α protein levels are downregulated and eIF4E-BP and PKR are dephosphorylated during FMDV infection (Ye et al., 2018). Ye et al. showed that G3BP-1 cleavage by 3C protease impairs SG assembly (Ye et al., 2018) while Visser et al. argued that L protease catalytic activity is responsible for the impairment of SG assembly in infected cells, without affecting PKR signaling (White et al., 2007). In addition, Ye et al. reported that the 3C-induced cleavage of G3BP-1 inhibits the NF-κB-dependent induction of antiviral immune responses (Ye et al., 2018). By using a reporter system, it has been shown that G3BP-1 negatively regulates viral translation by interacting with a structure located at domain 4 of the viral IRES (Galan et al., 2017). Furthermore, the G3BP-1 S149A substitution impairs the negative effect of G3BP-1 on IRES translation, suggesting that G3BP-1 is an antiviral protein whose activity depends on its phosphorylation (Ye et al., 2018). Instead, FMDV induces the nuclear-to-cytoplasm translocation of Sam68 via a proteolytic cleavage of its C-terminal domain mediated by 3C protease (Lawrence et al., 2012). Interestingly, Sam68 and TIA-1 colocalize in transient cytoplasmic granule-like structures in infected cells. Moreover, Sam68 interacts with FMDV IRES and Sam68 knockdown leads to a reduction in virus production, suggesting that Sam68 is a proviral factor (Lawrence et al., 2012; Rai et al., 2015). Similar to FMDV, Equine Rhinitis A virus (ERAV) infection also disrupts SG assembly via L-protease mediated cleavage of G3BP-1 and G3BP-2, suggesting that this is a conserved mechanism among aphtoviruses. However, despite G3BP-1 cleavage at multiple positions during FMDV and ERAV infections, the products differ in molecular weight, suggesting that they do not induce identical cleavages of G3BP-1 (Visser et al., 2019).

#### Togaviridae

Among viruses of *Togaviridae* family, Chikungunya virus (CHIKV) is the only member know to block SG assembly. G3BP-1 is sequestered by nsP3 in cytoplasmic foci (Fros et al., 2012) while G3BP-2 colocalizes with nsP2/3 in complexes different from viral replication factories (Scholte et al., 2015). Recently, it has been shown that dsRNA foci, nsP3-like granules and nsP1-coated structures are in close proximity, suggesting that CHIKV not only sequesters G3BP-1/-2 proteins in order to impair SGs assembly, but also to support viral replication (Remenyi et al., 2018). CHIKV dsRNA was shown to be undetectable in G3BP-1/-2 double knock-out (dKO) cells, indicating that G3BPs play key roles in RNA replication and formation of viral replication complexes (Kim et al., 2016). In contrast, Venezuelan Equine Encephalitis virus (VEEV) replication is not affected by G3BP-1/-2 dKO (Kim et al., 2016), but the SG-associated proteins FXR1, FXR2, and FMR1 have been shown to be essential factors for VEEV replication and protein production. Interestingly, VEEV infected cells contain both large and small plasma membrane-bound FXR-nsP3 complexes containing viral genomic RNA, suggesting a role of FXRs in viral replication and protection of viral genomic RNA from degradation during transport to the plasma membrane (Kim et al., 2016). Semliki Forest Virus (SFV) induces SG assembly early during infection in an eIF2α phosphorylation-dependent manner (McInerney et al., 2005). Nevertheless, at late stages of infection nsP3 promotes SG disassembly by sequestering G3BP-1 to sites of viral replication, which correlates with an increase in viral RNA levels (McInerney et al., 2005; Panas et al., 2012). Consistently, infection with a non-G3BP-1 binding SFV promotes a persistent accumulation of SGs containing G3BP-1 and TIA-1, which correlates with an attenuation in viral infection (Panas et al., 2015). On the other hand, Sindbis virus (SINV)-derived vectors induce PKR activation and the subsequent assembly of SGs containing TIA-1, eIF4E, and eIF4G (Venticinque and Meruelo, 2010). Furthermore, viral nsP2, nsP3, and nsP4 colocalize with aggregates containing G3BP-1 (Frolova et al., 2006; Gorchakov et al., 2008; Cristea et al., 2010) while nsP3 also interacts with G3BP-2. In 2011, Mohankumar et al. revealed that SINV infection induces the phosphorylation of eIF2α which correlates with a strong shutoff of *de novo* protein synthesis and 4E-BP1 dephosphorylation. Moreover, the authors demonstrated that SINV replication does not require the PI3K/Akt/mTOR pathway, and that later during infection, SINV suppresses Akt/mTOR activation in HEK cells (Mohankumar et al., 2011). Similar to CHIKV, G3BP-1/-2 dKO significantly reduce SINV replication rates and plaque size. However, FXR1/2 and FMR1 triple knock-out only induces a delay in viral particles production (Kim et al., 2016). Finally, it has been suggested that G3BP-1 plays a potential role in the encapsidation of Rubella virus (RUBV) due to the colocalization of RUBV genomic RNA, the non-structural viral protein P150 and G3BP-1 aggregates (Matthews and Frey, 2012).

#### Flaviviridae

West Nile virus (WNV), a member of the *Flaviviridae* family, was the first virus described to block SG assembly. The 3′ stem loop in the (–) RNA, which is the site of initiation for nascent genome RNA synthesis, captures the SG components TIA-1 and TIAR, suggesting that they have a role in viral replication (Li et al., 2002). In addition, TIA-1 and TIAR colocalize with viral replication complexes containing dsRNA and NS3 viral protein in the perinuclear region (Emara and Brinton, 2007). Although WT WNV impedes SGs assembly, the chimeric WNV W956IC induces PKR-dependent SG assembly due to the high levels of viral RNA that are produced (Courtney et al., 2012). Remarkably, WNV inhibits arsenite, but not heat shock, or DTT-induced SG assembly. High levels of GSH (antioxidant) has been shown to counteract arsenite-induced SGs, as during WNV infection even low levels of PERK-mediated eIF2α phosphorylation upregulate ATF4 and Nrf2, transcription factors that induce antioxidant gene expression (Basu et al., 2017). Similar to WNV, TIA-1, and TIAR colocalize with viral replication complexes containing dsRNA and NS3 in Dengue Virus type 2 (DENV-2) infected cells (Emara and Brinton, 2007). In addition, a quantitative mass spectrometry study revealed that DENV-2 RNA interacts with the SG components G3BP-1/2, Caprin1, and USP10 (Ward et al., 2011). It has been shown that DENV infection generates a non-coding subgenomic flaviviral RNA (sfRNA) that binds to G3BP-1/2 and Caprin1, impairing its ability to induce the translation of interferon Stimulated Genes (ISGs) mRNAs in response to DENV infection (Bidet et al., 2014). Recently, it has been described that Zika virus (ZIKV) infection blocks SG assembly (Amorim et al., 2017; Basu et al., 2017; Hou et al., 2017; Bonenfant et al., 2019) despite a strongly induced translational shutoff and activation of both PKR- and UPR-induced phosphorylation of eIF2α, suggesting that ZIKV impairs SG assembly downstream of eIF2α phosphorylation (Hou et al., 2017). In addition, ZIKV infection impairs arsenite-, poly I:C and hippuristanol, but not DTT-, Pateamine A- and Selenite-induced SG assembly (Amorim et al., 2017; Hou et al., 2017; Bonenfant et al., 2019) without affecting levels of SG-nucleating proteins (Amorim et al., 2017; Bonenfant et al., 2019). Hou et al. showed that expression of ZIKV NS3, NS4, NS2B-3 or capsid protein are sufficient to inhibit SG assembly (Hou et al., 2017). Interestingly, during ZIKV infection the host proteins YB-1 and Ataxin-2 are redistributed to the nucleus, while HuR and TIA-1 are redistributed to the cytoplasm of infected cells (Bonenfant et al., 2019). Moreover, TIAR is partially redistributed to sites of viral replication in the perinuclear zone, as seen on its colocalization with NS1 and viral RNA (Amorim et al., 2017). Furthermore, G3BP-1 and HuR are isolated with replication complexes, but only G3BP-1 interacts with viral dsRNA (Hou et al., 2017; Bonenfant et al., 2019). G3BP-1, Caprin-1, TIAR, Ataxin-2 and YB-1 knockdown negatively affects virus production, while HuR and TIA-1 knockdown resulted in an increase of viral titers (Hou et al., 2017; Bonenfant et al., 2019). Specifically, G3BP-1 knockdown also decreases genomic RNA and viral protein levels, while HuR knockdown increases genomic RNA and protein level (Bonenfant et al., 2019). Together, these data suggest a possible proviral role of the SG components G3BP-1, Caprin-1, TIAR, Ataxin-2, and YB-1 in ZIKV replication (Hou et al., 2017; Bonenfant et al., 2019). Similar to ZIKV, Japanese encephalitis virus (JEV), Murray Valley Encephalitis Virus (MVEV) and Yellow Fever Virus (YFV) capsid-expressing cells showed a significantly impairment in hippuristanol-induced SG assembly (Hou et al., 2017). Specifically, it has been shown that JEV core protein blocks SG assembly through an interaction with Caprin-1, resulting in the recruitment of other SG components such as G3BP-1 and USP10 (Katoh et al., 2013). A JEV virus carrying a non Caprin-1-binding core protein is less pathogenic in mice and exhibits lower propagation *in vitro* than WT virus, suggesting that SGs blockade is crucial to facilitate viral replication (Katoh et al., 2013). Analogous to WNV and DENV, Tick-Borne Encephalitis virus (TBEV) sequesters TIA-1 and TIAR to viral replication factories (Albornoz et al., 2014). In particular, TIA-1 binds viral RNA and acts as a negative regulator of TBEV translation, suggesting that TIA-1 function is independent of SG assembly (Albornoz et al., 2014). In addition, TBEV infection induces eIF2α-dependent SG assembly containing the canonical SGs markers G3BP-1, eIF3, and eIF4B (Albornoz et al., 2014). On the other hand, Bovine Viral Diarrhea virus (BVDV) impairs the assembly of arsenite-induced SGs and despite viral N-terminal protease (Npro) interaction with several SG components (such as YB-1, IGFBP2, DDX3, ILF2, and DXH9), this is not the mechanism by which BVDV blocks SG assembly (Jefferson et al., 2014). Hepatitis C virus (HCV) relocalizes G3BP-1, PABP1, ATX2, DDX3, TIA-1, and TIAR to viral replication factories in lipid droplets (LDs) (Ariumi et al., 2011; Garaigorta et al., 2012). In particular, DDX3 activates IKKα during HCV infection to induce LDs biogenesis (Li et al., 2013). Importantly, Garaigorta et al. reported that G3BP-1, TIA-1, and TIAR are required for viral RNA and protein synthesis early during infection, while G3BP-1, DDX3, and TIA-1 play a role in viral particle assembly (Garaigorta et al., 2012; Pène et al., 2015; Valiente-Echeverría et al., 2015). In addition, they showed that HCV induces SG assembly in a PKR-dependent manner in order to impair the translation of antiviral ISGs (Garaigorta et al., 2012). Ruggieri et al. showed that HCV induces an oscillation between SG assembly and disassembly as a result of balance between dsRNA-dependent PKR activation with the subsequent phosphorylation of eIF2α and the antagonist effect of GADD34-mediated dephosphorylation of eIF2α (Ruggieri et al., 2012). This tight balance allows HCV to chronically infect cells without affecting cell survival (Ruggieri et al., 2012). In addition, two other SG components have been related to HCV replication: Staufen1 and YB-1. Staufen1 is involved in cellular mRNA transport, translation and decay, and negatively regulates the assembly of SGs (Thomas et al., 2009). Despite YB-1 being a general translational repressor, it regulates SG assembly by inducing G3BP-1 mRNA translation through its interaction with the 5′UTR of the mRNA (Somasekharan et al., 2015). In 2016, Dixit et al. showed that Staufen1 interacts directly with PKR and NS5B, and that this interaction is required to inhibit PKR activation during HCV infection to allow viral RNA translation. In addition, the interaction of Staufen1 with NS5B suggests a role of Staufen1 in HCV replication, which is in accordance with a strong reduction in viral RNA and NS5A and NS5B protein levels in cells transfected with and Staufen1-siRNA (Dixit et al., 2016). Moreover, Wang et al. demonstrated that YB-1 knockdown reduces the phosphorylation status of NS5A, which is crucial for the NS5A-mediated regulation of RNA replication and virus assembly (Wang et al., 2015). Also, YB-1 interacts with NS5A in an YB-1 phosphorylation-dependent manner and this interaction is crucial for NS5A protein stability during HCV infection. Interestingly, YB-1 is phosphorylated by Akt at serine 102 and is known that HCV infection and NS5A expression activate the PI3K/Akt signaling (Wang et al., 2015). Together, these observations could explain the oscillation of SG assembly/disassembly detected in HCV-infected cells (Ruggieri et al., 2012) and how SG assembly and SG components are necessary for HCV RNA replication, assembly and egress (Ariumi et al., 2011; Garaigorta et al., 2012; Pager et al., 2013).

#### Dicistroviridae

Cricket Paralysis Virus (CrPV) is the only described member of *Dicistroviridae* family that regulates SG assembly. CrPV 1A protein impairs the assembly of arsenite-, Pateamine A-, and heat shock-induced SGs containing Rox8 and Rin, *Drosophila* homologs of TIA-1 and G3BP-1 respectively, demonstrating that there is a conserved mechanism in insect and human cells (Khong and Jan, 2011; Khong et al., 2016). In addition, CrPV-induced inhibition of SG assembly is not due to a cleavage of Rox8 or Rin despite 3C proteinase sequestration in SGs (Khong and Jan, 2011).

#### *Coronaviridae* and *Arteriviridae*

Transmissible gastroenteritis virus (TGEV), a member of the *Coronaviridae* family, induces SG assembly later during infection (Sola et al., 2011). The SGs component PTB binds to TGEV genomic and subgenomic RNA and colocalize with TGEV-induced aggregates containing TIA-1 and TIAR (Sola et al., 2011). In addition, Xue et al. described that PERK-mediated eIF2α phosphorylation during TGEV infection is detrimental for viral replication due to the global translational repression induced by activation of the IFN pathway (Xue et al., 2018). On the other hand, Mouse Hepatitis Coronavirus (MHV) induces the aggregation of TIAR early during infection in an eIF2α phosphorylation-dependent manner and, in contrast to TGEV, translational shutoff induced by MHV enhanced viral replication (Raaben et al., 2007). Moreover, MHV infection does not induce the expression of factors necessary to dephosphorylate eIF2α such as CHOP and GADD34 (Bechill et al., 2008). MHV N protein strongly impairs the IFN-induced PKR signaling activation, suggesting a viral regulation of the cellular antiviral response (Ye et al., 2007). Recently, Middle East Respiratory Syndrome Coronavirus (MERS-CoV) was shown to impair SG assembly even when viral dsRNA alone activates PKR-mediated SG assembly, suggesting that the virus protects its viral dsRNA from PKR (Rabouw et al., 2016; Nakagawa et al., 2018). Rabouw et al. showed that viral protein p4a antagonizes PKR activity through its dsRNA-binding motif and inhibits partially arsenite-dependent SG assembly, suggesting that p4a suppresses PKR but no other pathways of the cellular stress response (Rabouw et al., 2016). In addition, MERS-CoV replication is significantly impaired in cells depleted of TIA-1 or G3BP-1/-2, suggesting a potential proviral role of these SG components (Nakagawa et al., 2018). Severe Acute Respiratory Syndrome Coronavirus (SARS-CoV) induces a strong inhibition of host protein synthesis mediated by the nsp1 viral protein, which interacts with the 40S ribosomal subunit, impairing 80S formation (Narayanan et al., 2008; Kamitani et al., 2009). In addition, SARS-CoV infection induces PKR-mediated eIF2α phosphorylation, while GCN2 protein levels decreased in infected cells (Krahling et al., 2008). Finally, Infectious Bronchitis Coronavirus (IBV) induces PERK and eIF2α phosphorylation at early times post infection, while induces GADD34 expression and the subsequent eIF2α dephosphorylation at late stages of the course of infection in order to maintain viral protein synthesis (Wang X. et al., 2009; Liao et al., 2013). Interestingly, *IfnB* mRNA, but not IFN protein was detected in the supernatant of IBV infected cells, probably due to a 5b-mediated inhibition of general protein synthesis (Kint et al., 2016). However, although it has been described that SARS-CoV and IBV regulate eIF2α phosphorylation in infected cells, it has not been evaluated whether it result in SG assembly or blockade. In contrast, it is known that Porcine Reproductive and Respiratory Syndrome Virus (PRRSV), a member of the *Arteriviridae* family, induces canonical SG assembly mediated by PERK and eIF2α phosphorylation in infected cells (Zhou et al., 2017).

#### Caliciviridae

Members of the *Caliciviridae* family block SG assembly by targeting G3BP-1. Although Feline Calicivirus (FCV) infection results in eIF2α phosphorylation, viral 3C-like NS6 proteinase cleaves G3BP-1, thus impeding SG assembly in infected cells (Humoud et al., 2016). Similarly, Murine Norovirus 1 (MNV1) induces a shutoff of global translation by triggering the phosphorylation of eIF4E and eIF2α in a PKR-dependent manner, without inducing SG assembly (Royall et al., 2015; Brocard et al., 2018; Fritzlar et al., 2019). Interestingly, MNV1 infected cells showed a redistribution of G3BP-1 to sites of viral replication closely to the nucleus, colocalizing with NS5 (Fritzlar et al., 2019) or NS3 viral protein (Brocard et al., 2018). Together, these observations showed that MNV impairs SG assembly by sequestering G3BP-1, thus, uncoupling the cellular stress response (Brocard et al., 2018; Fritzlar et al., 2019). Although Humoud et al. showed that MNV does not impair arsenite-induced SGs, recently Fritzlar et al. demonstrated the opposite (Humoud et al., 2016; Fritzlar et al., 2019).

### Negative-Sense Single Stranded ((–) ssRNA) Viruses

#### Orthomyxoviridae

Influenza A virus (IAV) and Influenza B virus (IBV), members of the *Orthomyxoviridae* family, block SG assembly during infection. IAV disrupts SGs accumulation by expressing three different proteins: the host-shutoff protein polymerase-acidic protein-X (PA-X), the nucleoprotein (NP), and the non-structural protein 1 (NS1) (Khaperskyy et al., 2014). PA-X inhibits SG assembly in an eIF2α-independent manner, and requires its endoribonuclease activity for this function (Khaperskyy et al., 2014). It causes nuclear relocalization of PABP1, a phenotype that has been observed with other viral host-shutoff proteins (Khaperskyy et al., 2014). In addition, it depletes poly(A) RNAs from the cytoplasm but promotes its accumulation in the nuclei (Khaperskyy et al., 2014). A recent publication demonstrates that PA-X selectivity degrades host RNAs by selecting transcripts that have undergone splicing, and that can interact with cellular proteins involved in RNA splicing (Gaucherand et al., 2019). NP can block arsenite-induced SGs accumulation in an eIF2α-independent manner, but its effect depends on its expression levels (Khaperskyy et al., 2014). In contrast, NS-1-mediated inhibition of SG assembly depends on the PKR pathway; NS-1 binding to dsRNA inhibits PKR autophosphorylation and subsequent eIF2α phosphorylation (Khaperskyy et al., 2011). Interestingly, the SGs-associated proteins RAP55, DDX3, and NF90 have been shown to interact with both NP and NS-1, which could represent the cell‘s attempt to inhibit IAV infection or most the virus hijacks these host proteins to block SG assembly (Wang P. et al., 2009; Mok et al., 2012; Li et al., 2016; Raman et al., 2016). NP and DDX3 are recruited to SGs during ΔNS1 IAV infection (Onomoto et al., 2012; Raman et al., 2016), but in presence of NS1 NP localizes to PBs instead, suggesting that NS1 is essential for NP escape from SGs (Mok et al., 2012). Normally NF90 leads to SG accumulation by directly binding and activating PKR, but in presence of IAV NS1, NF90 binds preferentially to it rather than PKR, suggesting that NS1 also suppress PKR activation by blocking NF90-PKR interaction (Wen et al., 2014; Li et al., 2016). Similarly, IBV requires NS1 in order to restrict SG assembly (Núñez et al., 2018). The vRNA sensor retinoic acid inducible gene I (RIG-I) is recruited to SGs and induces IFN response during ΔNS1 IAV and IBV infections (Onomoto et al., 2012; Núñez et al., 2018). Furthermore, RIG-I was shown to associate with DDX6, which upon binding to vRNA stimulated RIG-I IFN induction (Núñez et al., 2018).

#### Arenaviridae

Infection with Junin virus (JUNV), a member of the *Arenaviridae* family, inhibits SG assembly in mock and arsenite-treated cells by impairing eIF2α phosphorylation. To do so, the presence of either NP or glycoprotein precursor (GPC) is required, as they both block SGs accumulation when expressed individually in cells (Linero et al., 2011). Recently, JUNV NP was found to interact with PKR, G3BP-1, eIF2α, hnRNP A1, and hnRNP K (King et al., 2017), as well as with DDX3 (Loureiro et al., 2018). Upon infection, PKR expression increases but is targeted to viral replication factories (RFs) together with NP, G3BP-1, dsRNA, PKR, phosphorylated PKR, RIG-I, and MDA-5 (King et al., 2017; Mateer et al., 2018). Despite the high levels of PKR activation, JUNV fails to induce eIF2α phosphorylation, maybe due its sequestration to the RFs via NP (King et al., 2017). Lassa virus and lymphocytic choriomeningitis virus (LCMV) NPs also interact with G3BP-1, eIF2α, and DDX3 (King et al., 2017; Loureiro et al., 2018). PKR interaction with LCMV NP occurs but weakly than with JUNV NP, which is reflected in the lack of PKR upregulation and colocalization with NP, and the increased eIF2α phosphorylation level compared to JUNV infection (King et al., 2017).

#### Rhabdoviridae

The vesicular stomatitis virus (VSV), member of the *Rhabdoviridae* family, promotes eIF2α phosphorylation and downregulates the synthesis of cellular proteins while maintaining viral production (Dinh et al., 2012). Under these conditions, it forms aSGs that contain PCBP2, TIA-1 and TIAR, but no eIF3 or eIF4A. VSV RNA, phosphoprotein (P) and NP are also part of these atypical SG-like structures, whose induction requires ongoing viral protein synthesis and viral replication (Dinh et al., 2012). Interestingly, assembly of aSGs and *bona fide* arsenite-induced SGs can occur simultaneously, revealing that VSV infection suppresses the accumulation of *bona fide* antiviral SGs and utilize SGs-associated components for its own benefit (Dinh et al., 2012). In contrast, Rabies virus (RABV) effectively replicate in cells that assemble SGs upon infection (Nikolic et al., 2016). The observed SGs contain G3BP-1, TIA-1 and PABP, and their accumulation is dependent on PKR-induced eIF2α phosphorylation, suggesting that they are canonical SGs. Notably, PKR and TIA-1 depletion enhances viral replication, revealing that they have an antiviral effect but that is not strong enough to completely stop RABV infection. RABV-induced SGs locate adjacent to viral RFs. Interestingly, viral mRNA but no viral genomic RNA is transported from RFs to SGs, suggesting that RABV may be using SGs to modulate viral transcription and replication (Nikolic et al., 2016).

#### Paramyxoviridae

Respiratory Syncytial Virus (RSV), member of the *Paramyxoviridae* family, replicate in viral replication factories (RFs) which have been observed to interact with SGs. However, seemingly contradictory findings have been reported for RSV. Lindquist et al. showed that RSV replication induces SG assembly in ~10–25% of the infected cells, and that they enhance RFs formation and viral replication (Lindquist et al., 2010, 2011). SGs accumulation requires PKR activation, which induces eIF2α phosphorylation, however PKR depletion did not affect RSV replication (Lindquist et al., 2011). Contrastingly, Groskreutz et al. reported that RSV infection activates PKR but does not trigger eIF2α phosphorylation due to PKR sequestration by the RSV NP (Groskreutz et al., 2010). Two other groups reported that RSV induce SG aggregation in ~1% (Hanley et al., 2010) and ~5% (Fricke et al., 2012) of the infected cells, revealing that, in general, RSV inhibits their assembly. Sequestration to RFs of the O-linked *N*-acetylglucosamine transferase (OGT), a factor involved in SGs regulation, and the presence of the 5′ extragenic trailer sequence of the RSV genome have been associated with SGs suppression (Hanley et al., 2010; Fricke et al., 2012). Measles virus (MeV) infection does not induce SG assembly due to the PKR inhibitory effect of the viral accessory protein C (Okonski and Samuel, 2012). To block PKR autophosphorylation, MeV C protein requires the presence of the dsRNA binding protein and the SGs-component ADAR1, as WT MeV infection induce PKR activation and SGs accumulation in ADAR1 depleted cells (Toth et al., 2009; Li et al., 2012; Okonski and Samuel, 2012). Furthermore, infection with ΔC-MeV produces large amounts of dsRNA that activate PKR and induce SGs assembly, suggesting that the C protein may utilize ADAR1 to downregulate the viral dsRNA produced during replication (Pfaller et al., 2013). Both ADAR1 and C protein colocalize with SGs (Okonski and Samuel, 2012). Similar to RSV, Sendai virus (SeV) induces SGs accumulation in just a fraction of the cells (5–15%) and the 5′ trailer region of its sequence has been implicated in SG assembly prevention via interaction with TIAR (Iseni et al., 2002). Like MeV, the SeV C protein is required to impair SG assembly in control and arsenite-treated cells, although C protein expression alone is not able to do so (Yoshida et al., 2015). ΔC-SeV assembled SGs contain RIG-I and unusual viral RNA species. Recently, the effect on SG assembly of three more paramyxoviruses have been studied. Newcastle disease virus (NDV) infection trigger the canonical SGs assembly to arrest host mRNAs and boost viral replication (Oh et al., 2016; Sun et al., 2017). SG assembly is dependent on PKR/eIF2α phosphorylation and its suppression (by depleting TIA-1 or TIAR proteins) reduces viral protein synthesis but increases cellular protein synthesis (Sun et al., 2017). Accordingly, cellular mRNAs have been shown to be predominately recruited to SGs compared to viral mRNAs (Sun et al., 2017). Strikingly, RIG-I is also recruited to the assembled SGs, which induces IFN production as an antiviral response (Oh et al., 2016). Likewise, Mumps virus (MuV) infection promotes SG assembly dependent on PKR activation despite weak eIF2α phosphorylation (Hashimoto et al., 2016). PKR, G3BP-1 and TIA-1 depletion reduces MeV-SGs and increased IFN response, but did not alter viral titers, suggesting that MuV replication occurs independently of the presence or absence of SGs. Conversely, Human Parainfluenza Virus Type 3 (HPIV3) infection leads to assembly of SGs that seem to have a poor antiviral role, as it is able to replicate in presence of SGs although viral protein expression and particle production is improved when SG assembly is constrained by knockdown of PKR, G3BP-1 or expression of a non-phosphorylatable eIF2α (Hu et al., 2018). SG assembly is due eIF2α PKR-dependent phosphorylation triggered by viral mRNAs, which can be shielded, and therefore block SGs assembly, by HPIV3 RFs (Hu et al., 2018).

#### Bunyaviridae

Since our last review (Poblete-Durán et al., 2016), no new reports have been published on how members of the *Bunyaviridae* family modulate SG assembly. Briefly, Rift Valley fever virus (RVFV) infection inhibit SG assembly despite attenuation of the Akt/mTOR signaling pathway which leads to the arrest of cap-dependent translation (Hopkins et al., 2015). It has been shown that non-structural protein from the S segment of Orthobunyaviruses, Hantaviruses and Phleboviruses (Kohl et al., 2003; Jaaskelainen et al., 2009), as well as glycoprotein Gn and the capsid N protein from Hantaviruses (Alff et al., 2006; Cimica et al., 2014; Matthys et al., 2014), inhibit IFN response.

Interestingly, the Andes Hantavirus (ANDV) N protein inhibits PKR dimerization, but this lack of activation does not stop protein translation (Wang and Mir, 2014). In contrast, RVFV infection promotes a protein translation shutoff due to PKR degradation by NSs protein (Habjan et al., 2009; Ikegami et al., 2009).

#### Filoviridae

Ebola virus (EBOV), member of the *Filoviridae* family, inhibits the assembly of SGs and instead sequesters the SGs-associated proteins eIF4G, eIF3, PABP, and G3BP-1, but no TIA-1 into granules within the viral replication factories (Nelson et al., 2016). These inclusion-bodies (IB) granules do not require eIF2α phosphorylation, do not disassemble with cycloheximide, and do not block translation. Furthermore, arsenite, heat and hippuristanol can still induce *bona fide* SGs accumulation, suggesting that sequestration of SGs proteins in IB granules may be released upon stress. EBOV VP35 was found to be the protein that prevents the *bona fide* SG assembly late in infection, and its C-terminal domain is critical for this function (Le Sage et al., 2016). VP35-CTD contains an inhibition of interferon regulatory factor 3 (IRF3) domain that is responsible for blocking PKR activation during EBOV infection (Schumann et al., 2009). However, when expressed in sufficient high levels, VP35 can block arsenite-induced SGs without reducing the levels of eIF2α phosphorylation, suggesting that VP35 suppress SG assembly by using an alternative way to PKR (Le Sage et al., 2016). VP35 can interact with several SG-associated proteins such as G3BP-1, eIF3, and eEF2 and it is targeted to viral replication factories, suggesting that it may be blocking SG assembly by relocating SG constituents (Le Sage et al., 2016; Nelson et al., 2016).

### Single Strand RNA Retroviruses (ssRNA-RTs)

#### Retroviridae

Viruses belonging the *Retroviridae* family integrate its retrotranscribed (+)ssRNA into the host chromosomal DNA (Fields et al., 2013). The Human T-Lymphotropic Virus 1 (HTLV1) Tax protein blocks SG assembly by interacting with the SG components histone deacetylase 6 (HDAC6) (Legros et al., 2011) and USP10 (Takahashi et al., 2013). Similarly, the Human Immunodeficiency Virus Type I (HIV-1) also blocks SG assembly despite eIF2α phosphorylation, through an interaction between Gag and the eukaryotic elongation factor 2 (eEF2) (Valiente-Echeverría et al., 2014; Poblete-Durán et al., 2016). HIV-1 Gag also disassembles pre-formed arsenite-induced SGs by interacting with G3BP-1 (Valiente-Echeverría et al., 2014) and selenite-induced atypical SGs by interacting with eIF4E (Cinti et al., 2016; Poblete-Durán et al., 2016). Notably, G3BP-1 was shown to act as a restriction factor that inhibits viral replication by interacting with HIV-1 genomic RNA (gRNA) in macrophages (Cobos Jiménez et al., 2015). Recently, Rao et al. described a novel HIV-1 NC-induced SGs containing G3BP-1, TIAR, eIF3, PABP, and poly(A) mRNAs that are no longer disassembled by Gag or CA (Rao et al., 2017). HIV-1 NC expression significantly increased eIF2α phosphorylation, inhibiting protein synthesis and reducing viral particle production. In addition, NC was shown to interact with G3BP-1, TIAR, and Staufen1 even in absence of RNA. The inability of NC to assembly SGs in G3BP-1 depleted cells suggest that their interaction is required to promote NC-induced SG accumulation (Rao et al., 2017). The authors also showed that Staufen1 counteracts NC-induced PKR-dependent eIF2α phosphorylation and translational shutoff (Rao et al., 2017). Interestingly, while HIV-1 impairs SGs assembly, the virus favors the assembly of Staufen1-containing HIV-1 dependent ribonucleoproteins (Abrahamyan et al., 2010). Indeed, it has been reported that HIV-1 is unable to dissociate or block arsenite-induced SG assembly in Staufen1 knock-out cells, suggesting that the recruitment of Staufen1 is crucial for HIV-1 SGs blockade. In addition, in absence of Staufen1 the HIV-1 genomic RNA colocalizes with TIAR in arsenite-induced SGs, which correlates with a significant reduction of Gag protein levels possibly due to the robust eIF2α phosphorylation induced by HIV-1 infection (Rao et al., 2019b). Besides these data, Soto-Rifo et al. showed that DDX3, eIF4GI, and PABPC1 form a pre-translation initiation complex with the HIV-1 genomic RNA to promote viral translation (Soto-Rifo et al., 2014; Poblete-Durán et al., 2016). In contrast to HIV-1, HIV-2 infection does not block SG assembly and the viral genomic RNA recruits TIAR in a different type of RNA granules where it is suggested that the transition from translation to packaging occurs (Soto-Rifo et al., 2014). On another hand, Bann et al. showed that Mouse Mammary Tumor Virus (MMTV) Gag interacts and colocalizes with the SGs component YB-1 in small cytoplasmic foci in an RNA-dependent manner. Interestingly, these foci also contain viral RNA and are insensitive to cycloheximide treatment. It is suggested that YB-1 plays key roles in MMTV as YB-1 knockdown results in a significant reduction in viral particle production (Bann et al., 2014).

In summary, information about virus-host interaction mediated by membraneless organelles in order to ensure viral replication can be found summarized in Table 2.

**Table 2:** Virus families that modulate SGs.

**Genome**	**Virus family**	**Virus**	**SGs induction**	**SGs blockade**	**Mechanism**	**References**
dsDNA	*Herpesviridae*	Herpes simplex Virus type 1 (HSV-1)	No	Yes	vhs is required for inhibition of SG assembly dependent on PKR	Esclatine et al., 2004; Dauber et al., 2011, 2016; Sciortino et al., 2013
					vhs-dependent SGs inhibition is independent on eIF2α phosphorylation	Dauber et al., 2011, 2016
					vhs-dependent SG inhibition is dependent on eIF2α phosphorylation, dsRNA partially localizes to SGs, and SG assembly activates PKR	Burgess and Mohr, 2018
					ICP27 inhibits phosphorylation of PKR/eIF2α and blocks SG assembly	Sharma et al., 2017
					ICP8 interacts with G3BP and blocks SG assembly	Panas et al., 2015
		Herpes simplex virus type 2 (HSV-2)	No	Yes	HSV-2 inhibits SG assembly independent on eIF2α phosphorylation	Finnen et al., 2012
					vhs is required for disruption of canonical and arsenite-induced SGs	Finnen et al., 2012
					vhs inhibit SG assembly and disrupt pre-assembled SGs, and its endoribonuclease activity is required	Finnen et al., 2016
		Cytomegalovirus (HCMV)	No	Yes	HCMV inhibits eIF2α phosphorylation	Isler et al., 2005b
					HCMV inhibits SG assembly	Isler et al., 2005a
					Lack of viral proteins pIRS1 and pTRS1 increase levels of eIF2α phosphorylation	Marshall et al., 2009
					pTRS1 or pIRS1 inhibits SG assembly dependent on PKR. Transfected pTRS1 also prevent SG assembly independent on PKR	Ziehr et al., 2016
		Kaposi's sarcoma-associated herpesvirus (KSHV)	No	Yes	ORF57 interacts with PKR inhibiting its binding to dsRNA and its activation, which impairs eIF2α phosphorylation and SG assembly	Sharma et al., 2017
					SOX inhibits arsenite-induced SG assembly	Sharma et al., 2017
		Epstein–Barr virus (EBV)	ND	ND	EB2 overexpression does not abolish SG assembly neither PKR/eIF2α phosphorylation	Sharma et al., 2017
	*Poxviridae*	Vaccinia virus (VACV)	Yes (AVGs)	Yes	G3BP-1, Caprin 1, eIF4G, eIF4E, PABP are sequestered into RFs	Katsafanas and Moss, 2004
					VACV lacking E3L induce AVGs which require TIA-1 expression	Simpson-Holley et al., 2010
					ΔE3L induced AVGs requires eIF2α phosphorylation	Pham et al., 2016
					WT VACV spontaneously form AVGs but to negligible levels	Rozelle et al., 2014
					ΔC7L/K1L VACV induce AVGs assembly dependent on SAMD9 host protein and independent of eIF2α phosphorylation	Liu and McFadden, 2014
					Viral mRNA is recruited to ΔC7L/K1L VACV induced AVGs	Sivan et al., 2018
					TIA-1 is not required for ΔC7L/K1L-mediated AVG assembly	Meng and Xiang, 2019
dsRNA	*Reoviridae*	Rotavirus	No	Yes	Rotavirus inhibits SG assembly independent on eIF2α phosphorylation, but changes the localization of TIA-1, eIF4E, and PABP	Montero et al., 2008
					Relocalizes ADAR1, Caprin1, CPEB, eIF2α, 4EBP1, PKR, and Staufen1 to RFs, and selectively excludes G3BP-1 and ZBP1	Dhillon and Rao, 2018; Dhillon et al., 2018
		Mammalian orthoreovirus (MRV)	Yes (canonical)	Yes	SGs are formed during the early stage of infection but disassembled in later stages independent on eIF2α phosphorylation	Qin et al., 2009, 2011
					μNS is recruited to SGs	Carroll et al., 2014
					MRV relocalizes G3BP-1, Caprin1, USP10, TIAR, TIA-1, eIF3b to RFs via G3BP1-oNS-uNS interaction	Núñez et al., 2018
(+)ssRNA	*Picornaviridae*	Poliovirus (PV)	Yes (canonical and atypical)	Yes	2A-protease mediated SGs assembly	Mazroui et al., 2006
					3C protease-mediated G3BP-1 cleavage	White et al., 2007
					Induces aggregates containing TIA-1 and viral RNA	Piotrowska et al., 2010
		Encephalomyocarditis virus (EMCV)	No	Yes	Cleavage of G3BP-1	Ng et al., 2013
		Coxsakievirus B3 CVB3	Yes (canonical and atypical)	Yes	Cleavage of G3BP-1	Fung et al., 2013
					2A protease-mediated SGs assembly	Zhai et al., 2018
					2A protease-mediated eIF4G cleavage	Wu et al., 2014
		Theiler's murine encephalomyelitis virus (TMEV)	No	Yes	Inhibition of SG assembly mediated by Leader protein (L)	Borghese and Michiels, 2011
		Enterovirus 71 (EV71)	Yes (canonical and atypical)	Yes	2A protease-mediated inhibition of SGs	Zhu et al., 2016; Yang et al., 2018b
					PKR-eIF2α phosphorylation- dependent SG assembly mediated by 2A protease	Zhang et al., 2016, 2018; Zhu et al., 2016
					Cleavage of eIF4GI mediated by 2A protease, abolishing eIF4GI and G3BP-1 interaction	(Yang et al., 2018a,b)
					Cleavage of G3BP-1 mediated by 3C protease	Zhang et al., 2018
		Foot-and-Mouth Disease Virus (FMDV)	No	Yes	Shuts-off host cap-dependent translation mediated by eIF2α downregulation and PKR dephosphorylation	Ye et al., 2018
					Cleavage of G3BP-1 and Sam68 mediated by 3C protease	Lawrence et al., 2012; Ye et al., 2018
					Cleavage of G3BP-1 mediated by Leader protease	Visser et al., 2019
		Equine Rhinitis A virus (ERAV)	ND	Yes	Cleavage of G3BP-1 and G3BP-2 by Leader protease	Visser et al., 2019
		Mengovirus, a strain of EMCV	No	Yes	Leader protein (L) inhibits SG assembly	Langereis et al., 2013; Reineke et al., 2015
	*Togaviridae*	Semliki Forest Virus (SFV)	Yes (canonical)	Yes	Induces eIF2α phosphorylation	McInerney et al., 2005
					nsP3 sequesters G3BP-1 and G3BP-2 into RFs	Panas et al., 2012
					G3BP-1 binding by nsP3 is necessary for SGs blockage	Panas et al., 2015
		Chikungunya virus (CHIKV)	No	Yes	Nsp3 sequesters G3BP-1 to RFs	Fros et al., 2012; Remenyi et al., 2018
					G3BP-2 colocalizes with nsP2/nsP3 complexes	Scholte et al., 2015
		Rubella virus (RUBV)	Yes	No	Accumulation of G3BP-1	Matthews and Frey, 2012
		Venezuelan equine encephalitis virus (VEEV)	ND	ND	nsP3 interacts with FXRs to facilitate viral RFs formation	Kim et al., 2016
		Sindbis virus (SINV)	Yes (canonical)	Yes	Nsp4 interacts with G3BP-1	Cristea et al., 2010
					Induces PKR-dependent SGs assembly	Venticinque and Meruelo, 2010
					Nsp3 protein interacts with G3BP-1 and G3BP-2	Kim et al., 2016
	*Flaviviridae*	West Nile Virus (WNV)	No	Yes	Viral RNA captures TIA-1 and TIAR	Li et al., 2002
					Increased GSH levels inhibit arsenite-induced SGs	Basu et al., 2017
		Dengue Virus (DENV)	No	Yes	Viral RNA colocalizes with TIA-1 and TIAR	Emara and Brinton, 2007
					3′UTR interacts with G3BP-1, G3BP-2, Caprin 1 and USP1	Ward et al., 2011
		Zika Virus (ZIKV)	No	Yes	ZIKV impairs SG assembly downstream of eIF2α phosphorylation	Hou et al., 2017
					Expression of ZIKV capsid, NS3, NS2B-3, or NS4A protein inhibits SG assembly. Capsid protein interacts with G3BP-1 and Caprin-1	Hou et al., 2017
					Relocalizes Ataxin-2, HuR and YB-1. G3BP-1 and TIAR localize at viral RFs	Bonenfant et al., 2019
		Yellow Fever Virus (YFV)	ND	Yes	Ectopically expressed capsid protein blocks hippuristanol-induced SGs	Hou et al., 2017
		Murray Valley Encephalitis Virus (MVEV)	ND	Yes	Ectopically expressed capsid protein blocks hippuristanol-induced SGs	Hou et al., 2017
		Tick-borne encephalitis virus (TBEV)	Yes (canonical)	No	Induces eIF2α phosphorylation	Albornoz et al., 2014
					Sequesters TIA-1 and TIAR to RFs	Albornoz et al., 2014
		Japanese encephalitis virus (JEV)	No	Yes	Core protein interacts with Caprin 1	Katoh et al., 2013
					Ectopically expressed capsid protein blocks hippuristanol-induced SGs	Hou et al., 2017
		Bovine viral diarrhea virus (BVDV)	No	Yes	Impairs arsenite-induced SGs assembly	Jefferson et al., 2014
		Hepatitis C virus (HCV)	Yes	Yes	G3BP-1, Ataxin-2, PABP1, DDX3, TIA-1, and TIAR are recruited to lipid droplets	Ariumi et al., 2011; Garaigorta et al., 2012
					Induces SGs dependent on PKR and IFN	Garaigorta et al., 2012
					GADD34-mediated SGs disassembly	Ruggieri et al., 2012
					DDX3 binds viral 3'UTR	Li et al., 2013
					DDX3 and G3BP-1 localize with HCV core protein	Pène et al., 2015
					Staufen 1 inhibits PKR activation	Dixit et al., 2016
	*Dicistroviridae*	Cricket paralysis virus (CrPV)	No	Yes	3C protease is sequestered to SGs	Khong and Jan, 2011
					CrPV-1A protein disrupts Pateamine A, arsenite and heat shock-induced SGs assembly	Khong et al., 2016
	*Coronaviridae*	Mouse hepatitis coronavirus (MHV)	Yes	No	Induces eIF2α phosphorylation	Raaben et al., 2007; Bechill et al., 2008
					N protein impairs PKR and eIF2α phosphorylation during IFN treatment	Ye et al., 2007
		Transmissible gastroenteritis virus (TGEV)	Yes	No	PTB localization in SGs correlates with an increase in viral replication	Sola et al., 2011
					Induces PERK-dependent eIF2α phosphorylation	Xue et al., 2018
		Middle East Respiratory Syndrome Coronavirus (MERS-CoV)	No	Yes	4a protein inhibits PKR-dependent SG assembly by binding and sequestering dsRNAs from PKR	Rabouw et al., 2016; Nakagawa et al., 2018
		Severe Acute Respiratory Syndrome Coronavirus (SARS-CoV)	ND	ND	Nsp1 induces translational shutoff by impairing 80S formation	Narayanan et al., 2008; Kamitani et al., 2009
					Induces PKR and eIF2α phosphorylation	Krahling et al., 2008
		Infectious Bronchitis Coronavirus (IBV)	ND	ND	Induces PKR and eIF2α phosphorylation at early stages of infection and inhibits eIF2α phosphorylation at later stages	Wang X. et al., 2009; Liao et al., 2013
					Viral 5b protein induces host translational shutoff	Kint et al., 2016
	*Arteriviridae*	Porcine Reproductive and Respiratory Syndrome Virus (PRRSV)	Yes (canonical)	ND	Induces PERK-dependent eIF2α phosphorylation and subsequent SGs assembly	Zhou et al., 2017
					Induces Mnk1-mediated eIF4E phosphorylation	Royall et al., 2015
					Induces PKR and eIF2α phosphorylation, but translation repression is PKR-independent	Fritzlar et al., 2019
					G3BP-1 is sequestered to RFs even in presence of arsenite treatment	Fritzlar et al., 2019
					G3BP-1 colocalizes with Nsp3 in a perinuclear zone but not in presence of arsenite treatment	Brocard et al., 2018
		Feline Calicivirus (FCV)	No	Yes	NS6-mediated G3BP-1 cleavage	Humoud et al., 2016
(-)ssRNA	*Orthomyxoviridae*	Influenza A virus (IAV)	No	Yes	NS1 restrict SG assembly dependent on eIF2α while NP and PA-X block SG assembly in an eIF2α-independent manner	Khaperskyy et al., 2014
					PA-X requires its endoribonuclease activity to inhibit SGs, and relocalizes PABP1 to the nucleus	Khaperskyy et al., 2014
					PA-X selectively degrades host spliced RNAs	Gaucherand et al., 2019
					NS-1 inhibits PKR activation by binding to dsRNA	Khaperskyy et al., 2011
					NS1 interacts with RAP55 and its RNA and PKR binding sites are required for the interaction and to inhibit SGs	Mok et al., 2012
					NS1 and NP interact with DDX3	Raman et al., 2016
					NP and RIG-I are recruited to SGs on ΔNS1 IAV infection, and IAV genomic RNA is sufficient to form SGs	Onomoto et al., 2012
					NS1 interacts with NF90 and restricts its binding to PKR. The NS1 RNA-binding domain and the NF90 double-stranded RNA binding domain are required	Wen et al., 2014; Li et al., 2016
					NF90 binds NP independently of RNA binding	Wang P. et al., 2009
		Influenza B virus (IBV)	No	Yes	NS1 is required to inhibit SG assembly. RIG-I and DDX6 interact and colocalize to ΔNS1-induced SGs	Núñez et al., 2018
	*Arenaviridae*	Junin Virus (JUNV)	No	Yes	NP and GPC individually impair arsenite-induced SGs by inhibiting eIF2α phosphorylation	Linero et al., 2011
					NP interacts with G3BP-1, PKR, hnRNP A1, and hnRNP K, G3BP-1 and eIF2α. NP sequesters PKR into RFs	King et al., 2017
					NP interacts with DDX3	Loureiro et al., 2018
					dsRNA activates PKR and colocalizes with RFs	Mateer et al., 2018
	*Rhabdoviridae*	Vesicular stomatitis virus (VSV)	Yes (atypical)	Yes	Inhibit canonical SGs but induces SGs-like structures containing PCBP2, TIA1, TIAR, and viral RNA, P and NP proteins	Dinh et al., 2012
		Rabies virus	Yes (canonical)	No	SG assembly is dependent on PKR and they locate close to RFs. Viral mRNA is transported to RFs	Nikolic et al., 2016
	*Paramyxoviridae*	Respiratory Syncytial Virus (RSV)	Yes (canonical)	Yes	10–25% of infected cells form SGs dependent on PKR	Lindquist et al., 2010, 2011
					Just 1% of infected cells form SGs. The 5‘trailer region of the RSV genome is required to inhibit SGs	Hanley et al., 2010
					Just 5% of infected cells form SGs. Sequestration of OGT into RFs suppresses SGs accumulation	Fricke et al., 2012
		Measles virus (MeV)	No	Yes	Viral C protein inhibits SG assembly by blocking PKR activation but requires the presence of ADAR1	Okonski and Samuel, 2012
					MeV C protein reduces the dsRNA in the cytoplasm to inhibit PKR activation	Pfaller et al., 2013
		Sendai virus (SeV)	Yes (canonical)	Yes	5–15% of infected cells form SGs. The trailer RNA region captures TIAR and inhibit SGs accumulation	Iseni et al., 2002
					Viral C protein is required to inhibit SG assembly	Yoshida et al., 2015
		Newcastle disease virus (NDV)	Yes (canonical)	No	NDV replication induces canonical SGs which contain vRNA(+) and RIG-I	Oh et al., 2016
					SG assembly is dependent on PKR/eIF2α pathway. SGs contain cellular mRNA but no viral mRNA	Sun et al., 2017
		Mumps virus (MuV)	Yes (canonical)	No	SG assembly is dependent on PKR. MuV replicates independently of the presence or absence of SGs	Hashimoto et al., 2016
		Human parainfluenza virus type 3 (HPIV3)	Yes	No	PKR-dependent SGs are induced by viral mRNA. SGs have an inhibitory role in HPIV3 replication.	Hu et al., 2018
	*Bunyaviridae*	Rift Valley fever virus (RVFV)	Yes	Yes	Attenuate Atk/mTOR signaling	Hopkins et al., 2015
		Andes hantavirus (ANDV)	ND	Yes	N protein inhibits PKR activation	Wang and Mir, 2014
	*Filoviridae*	Ebola virus	Yes (IB granules)	Yes	Ebola inhibits canonical SGs but form IB granules within RFs that contain eIF4G, eIF3, PABP, and G3BP-1, but no TIA-1	Nelson et al., 2016
					VP35 inhibit canonical and stress-induced SGs, and its C-terminal domain is required. VP35 interacts with G3BP-1, eIF3, and eEF2	Le Sage et al., 2016
ssRNA-RT	*Retroviridae*	Human T-cell Leukemia virus (HTLV-1)	No	Yes	Tax interacts with HDAC6 and USP10	Legros et al., 2011; Takahashi et al., 2013
		Human immunodeficiency virus type 1 (HIV-1)	No	Yes	Staufen 1 and Gag-mediated blockade of SGs assembly	Abrahamyan et al., 2010
					Gag interacts with eEF2 to block SGs assembly	Valiente-Echeverría et al., 2014
					G3BP-1 interact with Gag to disassembly preformed SGs	Valiente-Echeverría et al., 2014
					gRNA promote pre-translation initiation complex assembly	Soto-Rifo et al., 2014
					Gag interacts with eIF4E to promote disassembly of SGs	Cinti et al., 2016
					Ectopically expressed NC protein induces eIF2α phosphorylation and interacts with SGs proteins.	Rao et al., 2017
		Human immunodeficiency virus type 2(HIV-2)	Yes	No	gRNA and TIAR colocalizes in SGs	Soto-Rifo et al., 2014
		Mouse Mammary Tumor Virus (MMTV)	ND	ND	YB-1 interacts with Gag and gRNA in cytoplasmic foci	Bann et al., 2014

## Viral Infections and Processing Bodies

PBs are membraneless organelles and their diameter range between 150 and 340 nm (Cougot et al., 2012). PBs contain proteins involved in mRNA decapping machinery (Dcp1/2, LSm1-7, Edc3 proteins), scaffolding proteins (GW182, Ge-1/Hedls), deadenylation factors (Ccr1, Caf1, Not1), nonsense-mediated decay (NMD) proteins (SMG5-6-7, UPF1) and translation control factors (CPEB, eIF4E-T) (reviewed in Poblete-Durán et al., 2016). PBs are constitutively assembled in the cytoplasm of the cell, but their size and number increases upon cellular stress (Kedersha et al., 2005). Initially it was suggested that PBs were sites of mRNA decay, but recent evidences suggest that instead, P-bodies are sites of long-term mRNA storage and decapping enzymes (reviewed in Luo et al., 2018). Recent studies have shown that PBs participate in the storage of a selection of mRNAs; transcripts involved in regulatory processes are enriched while mRNAs that encode proteins that support basic cell functions are excluded (Hubstenberger et al., 2017; Standart and Weil, 2018).

Interestingly, viruses have been shown to modulate PB assembly by degrading and/or relocating PB-associated components, thus avoiding their accumulation. Here we summarize how viruses modulate PBs.

### Double-Stranded DNA (dsDNA)Viruses

#### Adenoviridae

In order to accumulate late mRNAs, adenovirus decreases the number of PBs in the cell by relocalizing several PB components, such as DDX6, LSm1, Ge-1, Ago2, and Xrn1 to aggresomes, where proteins are inactivated or degraded (Greer et al., 2011). Aggresome formation is induced by the viral protein E4 11K, which was found to specifically bind DDX6, suggesting that this interaction may redistribute DDX6 (Greer et al., 2011). Recently, the PB-associated protein PatL1 was also shown to localize within aggresomes following E4 11K protein expression (Friedman and Karen, 2017).

#### Herpesviridae

In contrast to the aforementioned adenovirus, expression of PB-associated proteins and PBs accumulation increase upon HCMV infection, but viral mRNA is not targeted to them, suggesting that HCMV mRNAs escape translation repression (Seto et al., 2014). HCMV-induced PB assembly requires cellular but no viral RNA synthesis and was independent of the translational status of the cell (Seto et al., 2014). KSHV prevents PB assembly during latent and lytic infection thanks to the activation of the cytoskeletal regulator RhoA GTPase (RhoA) (Corcoran et al., 2012, 2015). How RhoA disperse PBs is unknown, but in both cases its activated by the p38/MK2 pathway, which is triggered by the viral gene G-protein-coupled receptor (vGPCR) during lytic infection, and by the viral kaposin B (KapB) protein during latency (Corcoran et al., 2012, 2015). Furthermore, on both cycles PBs inhibition causes stabilization of AU-rich containing RNAs, a *cis-acting* RNA element usually present in mRNAs coding cytokines, growth factors and proto-oncogenes, increasing their protein synthesis during infection (Corcoran et al., 2012, 2015).

### Double-Stranded RNA (dsRNA) Viruses

#### Reoviridae

Similarly, rotavirus has also been shown to suppress PB assembly (Montero et al., 2008; Bhowmick et al., 2015). Upon infection, it reduces the cytosolic levels of Xrn1, Pan3, and Dcp1a, but no GW182 in a time-dependent manner (Bhowmick et al., 2015). Furthermore, it has been shown that rotavirus infection induces the relocalization of Xrn1, Dcp1a, and PABP to the nucleus (Montero et al., 2008; Bhowmick et al., 2015), and to sequester most of the PB components into RFs, with the exception of DDX6, Edc4, and Pan3 (Dhillon and Rao, 2018; Dhillon et al., 2018), and to accelerate Pan3 decay by the NSP1 protein (Bhowmick et al., 2015).

### Positive-Sense Single Stranded RNA ((+) ssRNA) Viruses

#### Flaviviridae

The relationship between (+)ssRNA and PB components has been extensively studied. Members of the Flavivirus genus of the *Flaviviridae* family generates a subgenomic flavivirus RNA (sfRNA) as a product of genomic RNA incomplete degradation by the exonuclease Xrn1 (Pijlman et al., 2008; Silva et al., 2010). In that process, Xrn1 stalls on highly structured sequences in the 3′ UTR, thus inhibiting Xrn1 activity and resulting in the accumulation of uncapped cellular mRNAs (Pijlman et al., 2008; Moon et al., 2012). Interestingly, sfRNA colocalizes with Xrn1 in PBs and is essential for viral-mediated cytopathogenesis (Pijlman et al., 2008). The flavivirus WNV reduces PB assembly during the course of infection through the sequestration of several PB components such as LSm1, GW182, Xrn1, DDX3, and DDX6 to viral replication factories (RFs) (Chahar et al., 2013). Similarly, DENV infection reduces PBs accumulation through an interaction of DENV 3′UTR with DDX6 (Ward et al., 2011). In addition, it has been shown that Dcp1b colocalizes with viral dsRNA, suggesting that DENV RNA replication occurs within PBs (Dong et al., 2015). LSm1, another PBs component, interacts with DENV RNA both *in vitro* and *in vivo* during DENV-2 infection, colocalizing with sites of viral replication (Dong et al., 2015). Moreover, downregulation of LSm1 negatively affects viral RNA accumulation and particle production, suggesting that LSm1 plays key roles in the early stages of the viral replicative cycle, such as translation and replication (Dong et al., 2015). In 2016 Balinsky et al. showed that NS4A and NS3 interact with IRAV, which is a constituent of PBs and is induced by DENV infection in an interferon-dependent manner. Interestingly, IRAV is relocalized to viral RFs in HEK-293T cells and in monocyte-derived macrophages (Balinsky et al., 2017). MOV10 is also relocalized to viral RFs, as shown by its colocalization with NS3. Both IRAV and MOV10 are restriction factors for DENV, as viral replication is significantly enhanced in KO and KD cells, respectively (Balinsky et al., 2017). ZIKV infection does not affect the morphology, localization or number of PBs per cell (Hou et al., 2017), however both DDX6 and DGCR8 are upregulated in ZIKV-infected neurospheres (Garcez et al., 2017). Interestingly, nonsense-mediated mRNA decay (NMD) transcripts are stabilized in ZIKV-infected cells, suggesting that NMD pathway is impaired in infected cells. In addition, ZIKV capsid interacts with several NMD components, including nuclear Upf1, which is targeted for proteasomal degradation (Fontaine et al., 2018). Downregulation of Upf1 prior to infection significantly increases RNA viral levels and consequently viral production, suggesting that Upf1 regulates early stages of ZIKV infection (Fontaine et al., 2018). Similar to the other flavivirus mentioned above, HCV induces the relocalization of several PB components such as DDX6, LSm1, Xrn1, PatL1, Ago2, Dicer, and DDX3 to sites of viral replication in lipid droplets (Ariumi et al., 2011; Berezhna et al., 2011; Pager et al., 2013) in order to promote viral replication (Ariumi et al., 2007, 2011; Berezhna et al., 2011; Pager et al., 2013). In particular, the decapping activators DDX6, LSm1, and PatL1 are crucial for the transition from translation to replication of HCV RNA (Scheller et al., 2009; Jangra et al., 2010). Although PB constituents play key roles during HCV infection, the presence of PBs is not necessary for efficient viral replication (Berezhna et al., 2011; Pérez-Vilaró et al., 2012). Analysis of liver biopsies from HCV-infected patients confirmed that HCV decreases the number of PB *in vivo* independent of the viral genotype, the inflammation status of the sample donor or whether the infection is chronical or recent. Interestingly and contrary to previous reports, DDX6 was shown to not be recruited to sites of viral replication at lipid droplets (Pérez-Vilar et al., 2015), although its role in HCV replication in patient samples remains to be elucidated. This article by Pérez-Vilaró et al. is the first evidence and confirmation of a direct relationship between a viral-induced pathogenesis and PB modulation. Furthermore, they reported differences in PB composition since DDX6 do not colocalize with Dcp1 in human hepatocytes *in vivo*, highlighting the potential variations between cell line-based experiments and analysis of human or animal models (Pérez-Vilar et al., 2015). Although HCV and BVDV do not generate a sfRNA from their 3′UTR as other flavivirus such as DENV, Moon et al. showed that Xrn1 stalls while attempting to degrade the 5′UTR of both HCV and BVDV, thus Xrn1 activity is repressed. This dysregulation causes a stabilization of mRNAs encoding proteins involved in innate immune responses and transcription factors, which have short half-life in uninfected cells (Moon et al., 2015). In addition, they suggest that HCV-mediated Xrn1 repression induces a feedback mechanism that prevents the initial steps of 5′-3′ decay, such as deadenylation and decapping (Moon et al., 2015).

#### Picornaviridae

The picornaviruses PV and CVB3 3C proteases cleave Dcp1a and target Xrn1 and Pan3 for proteasomal degradation, thus resulting in the total disruption of PBs (Dougherty et al., 2011; Poblete-Durán et al., 2016). In addition, CVB3 2A protease relocalizes AUF1 (also known as hnRNP D) from the nucleus to the cytoplasm of infected cells and 3C protease cleaves AUF1 (Wong et al., 2013). Moreover, AUF1 knockdown significantly enhances viral RNA abundance, suggesting that AUF1 is a restriction factor for CVB3 replication. Interestingly, AUF1 binds to the 3′UTR of viral genome containing AU-rich sequences and likely targets it for degradation, thus CVB3 counteracts AUF1-induced degradation of the viral RNA genome by targeting AUF1 for degradation (Wong et al., 2013). In addition, it has been reported that MOV10 is a restriction factor for EMCV and CVB3 replication. Thus, both EMCV and CVB3 viral 3C protease induces the cleavage of MOV10 to counteract its antiviral activity (Cuevas et al., 2016). Enterovirus 71 (EV71) increases the number of PBs at early stages but disrupt PBs at late stages of infection (Wang et al., 2016; Zhu et al., 2016; Yang et al., 2018b). In addition, EV71 2C protease reduces the expression of APOBEC3G (A3G) by targeting to degradation via the autophagy-lysosome pathway. Surprisingly, A3G antiviral activity does not depend on its cytidine deaminase activity but restricts viral replication by binding to the viral 5′UTR thus displacing PCBP1, which is essential for the replication of picornaviruses such EV71 (Li et al., 2018). In contrast, MOV10 is a positive regulator of EV71 replication through its binding to a cloverleaf-like structure and the IRES of viral RNA, facilitating replication and viral protein synthesis (Wang et al., 2016). Upon EV71 infection MOV10 co-localized with PBs and it is suggested that MOV10 relocalization is a host response to impair viral MOV10 recruitment (Wang et al., 2016).

#### *Dicistroviridae* and *Togaviridae*

CrPV, a member of the *Dicistroviridae* family, selectively disrupts GW182/Dcp1 but not Ago1/Ago2 aggregates, suggesting that they play differential roles during infection (Khong and Jan, 2011), while that RNAs of SINV, a virus that belong to the *Togavirida****e*** family, interact with HuR in order to stabilize and avoid the cellular mRNA decay machinery (Sokoloski et al., 2010). It was reported that Upf1 knockdown increases SINV and Semliki Forest Virus (SFV) RNA replication. Thus, Upf1 has an antiviral activity, probably by promoting viral RNA degradation. In addition, Upf1-mediated inhibition of viral replication also involved other NMD components, such as Smg5 and Smg7 (Balistreri et al., 2014). On other hand, the coronavirus MHV induces the degradation of several cellular mRNAs encoding translation-related factors with a concomitant translational shut off and increase in the number of PBs per cell (Raaben et al., 2007). The mechanism behind MHV-induced regulation of mRNA decay is still unknown. Similarly, SARS-CoV Nsp1 promotes mRNA downregulation as a consequence of global mRNA degradation in order to maximize viral RNA translation (Huang et al., 2011). In contrast, TGEV infection decreases significantly the number of PBs by an unknown mechanism (Sola et al., 2011).

### Negative-Sense Single Stranded ((–) ssRNA) Viruses

#### *Orthomyxoviridae* and *Bunyaviridae*

The negative-strand RNA virus IAV, member of *Orthomyxoviridae* family, suppresses PB assembly via interaction of NS1 and RAP55; and prevents the sequestering of NP in the PBs (Mok et al., 2012). In contrast, the *Bunyaviridae* hantavirus nucleocapsid protein (N) avoids the 5′ cap degradation of cellular mRNAs, protecting them from Dcp1a/Dcp2-mediated decapping which allows the viral transcripts to escape from PBs (Mir et al., 2008) (reviewed in Poblete-Durán et al., 2016).

#### *Paramyxoviridae* and *Rhabdoviridae*

Recently, it was shown that the infection of RSV, member of *Paramyxoviridae* family, decreases the number of Dcp1-containing puncta late in infection, suggesting that RSV disassemble PBs over time or that Dcp1 is excluded from PBs (Dickey et al., 2015); while that during early stages of VSV infection, virus belong to *Rhabdoviridae* family, Dcp1/PBs accumulation is unaltered, but further work is necessary to determine its effect on later times (Dinh et al., 2012).

### Single Strand RNA Retroviruses (ssRNA-RTs)

#### Retroviridae

Abrahamyan et al. reported that HIV-1 expression dramatically decreases the number of PBs (Abrahamyan et al., 2010), but the relationship between HIV-1 and the PB components is still controversial. It has been shown that HIV-1 gRNA localize to PBs (Chable-Bessia et al., 2009; Nathans et al., 2009; Martin et al., 2011), however, that observation has not been reproduced by others (Abrahamyan et al., 2010; Phalora et al., 2012). Similarly, some groups have reported that DDX6, LSm1, GW182, Xrn1, DGCR8, Dicer, and Drosha are antiviral factors, while other researchers argue that Ago2 and DDX6 are proviral factors (Chable-Bessia et al., 2009; Nathans et al., 2009; Martin et al., 2011; Bouttier et al., 2012; Reed et al., 2012). APOBEC3 has been shown to have an anti-HIV-1 activity, but it is targeted for degradation by the viral protein Vif (Poblete-Durán et al., 2016). Interestingly, Chen et al. showed that MOV10 protects A3G from Vif-mediated degradation by interfering with the interaction between Vif and the ubiquitin CBF-β-Cullin 5-ElonginB-ElonginC complex (Chen et al., 2017). In addition, MOV10 increases the incorporation of A3G in HIV-1 viral particles, enhancing the antiviral effect of A3G (Chen et al., 2017). Recently, it has been shown HIV-1 infected monocyte-derived macrophages (MDMs) show diminished levels of Upf1, Upf2, and SMG6 proteins, accordingly with their restrictive role in HIV-1 replication due to their ability to inhibit genomic RNA expression (Rao et al., 2019a). Interestingly, Reed et al. demonstrated that the first assembly intermediate (AI) in which Gag interacts with genomic RNA—containing HIV-1 Gag, GagPol, and Vif (Lingappa et al., 1997)—are formed by the recruitment of DDX6 and ATP-binding cassette protein E1 (ABCE1), suggesting that HIV-1 hijack PB-components to promote viral assembly (Reed et al., 2012; Barajas et al., 2018). The Feline Immunodeficiency Virus (FIV) also co-opts a cellular RNA granule containing DDX6, ABCE1, and Dcp2 to assembly immature capsids, suggesting that this is a mechanism conserved between primate and non-primate lentiviruses (Reed et al., 2018). It has been reported that HTLV-1 Tax protein interferes with host NMD by its interaction with Upf1 and INT6 (Mocquet et al., 2012). In addition, Tax increases the localization of Upf1 in PBs, causing an increase in their size and number. Interestingly, viral RNA is sensitive to degradation via NMD and it has been shown that a fraction of viral genomic RNA co-localized with PBs (Mocquet et al., 2012; Nakano et al., 2013). Fiorini et al. reported that Tax stabilizes the SMG5 and Upf1 interaction, which inhibits Upf1 recycling for another round of NMD (Mocquet et al., 2012). Moreover, Tax inhibits Upf1 binding to its substrate and also destabilizes Upf1 during both unwinding and translocation, promoting its dissociation from the substrate. Together, these observations suggest that Upf1 is targeted by Tax both before and during Upf1-mediated decay (Fiorini et al., 2018). In contrast, Nakano et al. argued that Rex protein is the main protein responsible for NMD inhibition in HTLV-1 infected cells and that Tax effect on NMD is not as significant as the effect of Rex protein (Nakano et al., 2013).

A summary of how viruses modulate PBs can be found in Table 3.

**Table 3:** Virus families that modulate PBs.

**Genome**	**Virus family**	**Virus**	**PB induction**	**PB blockade**	**Mechanism**	**References**
dsDNA	*Adenoviridae*	Adenovirus	No	Yes	Relocalization of DDX6, LSm1, Ge-1, Ago2, and Xrn1 to aggresomes dependent on E4 11K viral protein	Greer et al., 2011
					Relocalization of Pat1b to aggresomes dependent on E4 11K viral protein	Friedman and Karen, 2017
	*Herpesviridae*	Cytomegalovirus (HCMV)	Yes	No	Increased Dcp1a, Edc4, DDX6, and Rap55 protein levels and PB accumulation, but viral mRNA is not sequestered	Seto et al., 2014
		Kaposi's sarcoma-associated herpesvirus (KSHV)	No	Yes	PB disruption during lytic replication requires RhoA activation, mediated by vGPCR activation pathway (vGPCR-MK2)	Corcoran et al., 2012
					PBs disruption during latency requires RhoA activation, mediated by Kaposin B activation pathway (KapB-MK2-hsp27-p11RhoGEF)	Corcoran et al., 2015
dsRNA	*Reoviridae*	Rotavirus	No	Yes	Xrn1, Dcp1, and Pan3, but not GW182 protein levels are reduced. NSP1 triggers Pan3 decay. Xnr1 and Dcp1 are translocated to the nucleus	Bhowmick et al., 2015
					PABP is relocalized to the nucleus dependent on the viral protein NSP3	Montero et al., 2008
					Most of PBs-associated proteins, except DDX6, Edc4, and Pan3, are recruited into RFs	Dhillon and Rao, 2018; Dhillon et al., 2018
(+)ssRNA	*Flaviviridae*	West Nile virus (WNV)	No	Yes	LSm1, GW182, DDX6, DDX3, and Xrn1 are sequestered to RFs	Emara and Brinton, 2007; Chahar et al., 2013
		Dengue Virus (DENV)	No	Yes	LSm1, GW182, DDX6, DDX3, MOV10, and Xrn1 are sequestered to RFs	Emara and Brinton, 2007; Chahar et al., 2013
					IRAV and MOV10 localizes to RFs and associates with DENV NS4A and NS3	Balinsky et al., 2017
					LSm1 binds to the Dengue virus RNA 3' UTR and localizes to viral RFs	Dong et al., 2015
		Zika Virus	No	No	DDX6 and DGCR8 are upregulated in ZIKV-infected neurospheres	Garcez et al., 2017
					ZIKV does not affects PBs abundance, morphology or localization	Hou et al., 2017
					ZIKV capsid protein specifically targets nuclear Upf1 for degradation via the proteasome	Fontaine et al., 2018
		Yellow fever virus (YFV)	Yes^*^	No	sfRNA co-localizes at PBs and inhibits Xrn1 activity	Silva et al., 2010
		Kunjin virus (KUNV), Australian strain of DENV	Yes^*^	No	sfRNA co-localizes at PBs and inhibits Xrn1 activity	Pijlman et al., 2008; Moon et al., 2012
		Hepatitis C virus (HCV)	Yes^*^	Yes	DDX6, LSm1, Xrn1, PATL1, Ago2, Dicer, and DDX3 localize to lipid droplets	Ariumi et al., 2011; Berezhna et al., 2011; Pager et al., 2013
					Dcp1 and GW182 not localize to viral factories	Pérez-Vilaró et al., 2012
					DDX6 did not colocalize at lipid droplets in hepatocytes from HCV-infected patients	Pérez-Vilar et al., 2015
					XRN1 stalls during exonucleolytic decay of the 5' UTRs of HCV	Moon et al., 2015
		Bovine Viral Diarrhea Virus (BVDV)	ND	ND	XRN1 stalls during exonucleolytic decay of the 5′ UTRs of BVDV	Moon et al., 2015
	*Picornaviridae*	Poliovirus (PV)	No	Yes	3C protease-mediated cleavage of Xrn1, Dcp1a, and Pan3	Dougherty et al., 2011
					Protease 2A inhibits PB assembly	Dougherty et al., 2015
		Coxsackievirus B3 (CVB3)	No	Yes	3C protease-mediated cleavage of Xrn1, Dcp1a, and Pan3	Dougherty et al., 2011
					Cytoplasmic redistribution and cleavage of AUF1, mediated by 2A and 3C protease, respectively	Wong et al., 2013
					Cleavage of MOV10 by 3C protease	Rao et al., 2019b
		Encephalomyocarditis virus (EMCV)	ND	ND	Cleavage of MOV10 by 3C protease	Cuevas et al., 2016
		Enterovirus 71 (EV-71)	Yes	Yes	Disrupts DDX6 and Dcp1a foci	Zhu et al., 2016; Yang et al., 2018b
					EV71 2C protein reduces the expression of A3G through autophagy–lysosome pathway	Li et al., 2018
					MOV10 promotes viral RNA replication and IRES-dependent translation	Wang et al., 2016
					MOV10 co-localizes with PBs upon EV71 infection	Wang et al., 2016
	*Dicistroviridae*	Cricket paralysis virus (CrPV)	Yes	Yes	Disrupts aggregates containing GW182 and Dcp1	Khong and Jan, 2011
	*Togaviridae*	Sindbis virus (SINV)	No	Yes	Viral RNA interacts with HuR	Sokoloski et al., 2010
					Upf1 is a restriction factor for SINV	Balistreri et al., 2014
		Semliki Forest Virus (SFV)	ND	ND	Upf1 is a restriction factor for SFV	Balistreri et al., 2014
	*Coronaviridae*	Mouse Hepatitis Coronavirus (MHC)	Yes	No	Induces host translational shutoff	Raaben et al., 2007
		Severe Acute Respiratory Syndrome Coronavirus (SARS-CoV)	ND	ND	SCoV nsp1-mediated promotion of host mRNA degradation	Kamitani et al., 2009; Huang et al., 2011
		Transmissible Gastroenteritis Virus (TGEV)	No	Yes	Decreases the number of PBs	Sola et al., 2011
(-)ssRNA	*Orthomyxoviridae*	Influenza virus A (IAV)	No	Yes	NS1 interacts with RAP55, Ago1, Ago2, and Dcp1a	Mok et al., 2012
	*Bunyaviridae*	Hanta virus	Yes^*^	No	Cap snatching occurs in PBs	Mir et al., 2008
	*Paramyxoviridae*	Respiratory Syncytial Virus (RSV)	ND	ND	Reduction of Dcp1 puncta over time	Dickey et al., 2015
	*Rabdhoviridae*	Vesicular stomatitis virus (VSV)	ND	ND	Dcp1 puncta are not affected	Dinh et al., 2012
ssRNA-RT	*Retroviridae*	Human Immunodeficiency virus type 1 HIV-1	ND	Yes	HIV-1 genomic RNA interacts with DDX6, Ago2, and APOBE3G	Nathans et al., 2009
					Relocalization of PBs out of zones where genomic RNA accumulates	Abrahamyan et al., 2010
					The first assembly intermediate where Gag interacts with viral RNA contains DDX6 and ABCE1	Reed et al., 2012; Barajas et al., 2018
					Overexpression of MOV10 inhibits HIV-1 replication	Burdick et al., 2010
					MOV10 inhibits the degradation of APOBEC3G through interference with the Vif-mediated ubiquitin–proteasome pathway	Dong et al., 2015
					Downregulation of Upf1, Upf2, and SMG6 in infected monocyte-derived macrophages	Rao et al., 2019a
		Feline Immunodeficiency Virus (FIV)	ND	ND	Assembly intermediates are formed by DDX6, Dcp2, and ABCE1	Reed et al., 2018
		Human T-cell lymphotropic virus type I (HTLV-1)	Yes	No	Tax inhibits NMD by targeting Upf1 and INT6	Mocquet et al., 2012; Fiorini et al., 2018
					Tax increases the localization of Upf1 in PBs	Mocquet et al., 2012
					A fraction of viral RNA colocalizes in PBs	Mocquet et al., 2012; Nakano et al., 2013
					Rex inhibits Upf1 activity	Nakano et al., 2013

* *Maintain PB endogenous*.

## Concluding Remarks

The knowledge about MLOs has increased exponentially during the last few years (reviewed in Guzikowski et al., 2019). Consequently, the historically proposed roles of SGs and PBs in mRNA storage and degradation have been challenged. SGs and PBs assembly have been shown to contribute to cell survival, inducing translational arrest and delay apoptosis by sequestration of RACK1 from JNK, while that SGs may inhibit growth signaling by diverting TORC1 from its active location at lysosomes (Arimoto et al., 2008; Kedersha et al., 2011; Thedieck et al., 2013; Arimoto-Matsuzaki et al., 2016). Single-molecule imaging studies have revealed that mRNAs can transiently interact or move between SGs and PBs (Wilbertz et al., 2018; Moon et al., 2019), and more interestingly, that mRNA translation and degradation dynamics are equivalent for MLO-engaged and “free” mRNAs, suggesting that their sequestration into granules does not regulate translation and decay (Wilbertz et al., 2018). Recently, Niewidok et al. revealed the existence of a relative immobile nanocore within SGs and a mobile outer liquid phase that allows a dynamic exchange of protein and mRNA between SGs (Niewidok et al., 2018). Importantly, the tight interaction between SG-nanocores and prion-like domains present in many SG and PB components has been proposed as a potential origin of neurotoxic protein aggregates that are linked to the progression of different neurodegenerative diseases (reviewed in Ramaswami et al., 2013; Dobra et al., 2018), growing MLOs as an attractive therapeutic target for the treatment of such syndromes. A similar interest is rising regarding MLOs and viral infections. As reviewed here, cellular and viral-induced MLOs have been associated with a spatiotemporal regulation of viral replication, viral assembly and host immune evasion (Schuster et al., 2018). MLOs allow the concentration of viral proteins and viral genome in the cytosol of infected cells, enabling a dynamic exchange and adaptation to changing environmental conditions. Despite all the information presented in this review about the modulation of the MLOs assembly, many molecular details of how some viral families proceed to subvert the MLOs remain unknown. Why some viruses promote or inhibit the MLO assembly is well-characterized. In many cases, these MLOs serve as viral factories recruiting cellular proteins to ensure efficient viral replication, while that the co-option of proteins involved in antiviral response (e.g., interferon-stimulated genes) could have a detrimental effect. On the other hand, the dynamism in the MLOs assembly/disassembly over the course of a viral infection would allow the chronicity of the infection (Ruggieri et al., 2012). Today, we know that not only proteins, but also RNA modifications have gained importance in the fate of mRNAs inside the cell. These RNA modifications (e.g., m6A, m5C) have been involved in triaging mRNAs to RNA granules (Anders et al., 2018) which have also been widely identified in the RNA genomes of several viruses such as HIV-1, ZIKV, HCV, IAV, KSHV, and SV40 (Reviewed in Pereira-Montecinos et al., 2017; Manners et al., 2018). Thus, the possibility to develop drugs that upregulate these pathways, the generation of molecules that disaggregate or target crucial viral -MLO interactions arises to impair viral replication (Jackrel et al., 2014). Furthermore, due to the shared mechanism that different viruses use to modulate MLOs, drugs targeting MLOs could eventually serve as broad-spectrum antivirals for infectious diseases.

## Author Contributions

All authors listed have made a substantial, direct and intellectual contribution to the work, and approved it for publication.

### Conflict of Interest

The authors declare that the research was conducted in the absence of any commercial or financial relationships that could be construed as a potential conflict of interest.

## References

[B1] Abrahamyan L. G. Chatel-Chaix L. Ajamian L. Milev M. P. Monette A. Clement J.-F.. (2010). Novel Staufen1 ribonucleoproteins prevent formation of stress granules but favour encapsidation of HIV-1 genomic RNA. J. Cell Sci. 123, 369–383. 10.1242/jcs.055897 20053637

[B2] Albornoz A. Carletti T. Corazza G. Marcello A. (2014). The stress granule component TIA-1 binds tick-borne encephalitis virus RNA and is recruited to perinuclear sites of viral replication to inhibit viral translation. J. Virol. 88, 6611–6622. 10.1128/jvi.03736-13 24696465 PMC4054376

[B3] Alff P. J. Gavrilovskaya I. N. Gorbunova E. Endriss K. Chong Y. Geimonen E.. (2006). The pathogenic NY-1 hantavirus G1 cytoplasmic tail inhibits RIG-I- and TBK-1-directed interferon responses. J. Virol. 80, 9676–9686. 10.1128/JVI.00508-06 16973572 PMC1617216

[B4] Amorim R. Temzi A. Griffin B. D. Mouland A. J. (2017). Zika virus inhibits eIF2α-dependent stress granule assembly. PLoS Negl. Trop. Dis. 11:e0005775. 10.1371/journal.pntd.0005775 28715409 PMC5531678

[B5] Anders M. Chelysheva I. Goebel I. Trenkner T. Zhou J. Mao Y.. (2018). Dynamic m6a methylation facilitates mRNA triaging to stress granules. Life Sci. Alliance 1, 1–12. 10.26508/lsa.201800113 30456371 PMC6238392

[B6] Arimoto K. Fukuda H. Imajoh-Ohmi S. Saito H. Takekawa M. (2008). Formation of stress granules inhibits apoptosis by suppressing stress-responsive MAPK pathways. Nat. Cell Biol. 10, 1324–1332. 10.1038/ncb1791 18836437

[B7] Arimoto-Matsuzaki K. Saito H. Takekawa M. (2016). TIA1 oxidation inhibits stress granule assembly and sensitizes cells to stress-induced apoptosis. Nat. Commun. 7, 1–10. 10.1038/ncomms10252 26738979 PMC4729832

[B8] Ariumi Y. Kuroki M. Abe K.-I. Dansako H. Ikeda M. Wakita T.. (2007). DDX3 DEAD-Box RNA helicase is required for hepatitis C virus RNA replication. J. Virol. 81, 13922–13926. 10.1128/jvi.01517-07 17855521 PMC2168844

[B9] Ariumi Y. Kuroki M. Kushima Y. Osugi K. Hijikata M. Maki M.. (2011). Hepatitis C virus hijacks P-body and stress granule components around lipid droplets. J. Virol. 85, 6882–6892. 10.1128/jvi.02418-10 21543503 PMC3126564

[B10] Aulas A. Fay M. M. Lyons S. M. Achorn C. A. Kedersha N. Anderson P.. (2017). Stress-specific differences in assembly and composition of stress granules and related foci. J. Cell Sci. 130, 927–937. 10.1242/jcs.199240 28096475 PMC5358336

[B11] Balinsky C. A. Schmeisser H. Wells A. I. Ganesan S. Jin T. Singh K.. (2017). IRAV (FLJ11286), an interferon- stimulated gene with antiviral activity against dengue virus, interacts with MOV10. J. Virol. 91:e01606-16. 10.1128/JVI.01606-16 27974568 PMC5309953

[B12] Balistreri G. Horvath P. Schweingruber C. Zünd D. McInerney G. Merits A.. (2014). The host nonsense-mediated mRNA decay pathway restricts mammalian RNA virus replication. Cell Host Microbe 16, 403–411. 10.1016/j.chom.2014.08.007 25211080

[B13] Bann D. V. Beyer A. R. Parent L. J. (2014). A murine retrovirus Co-Opts YB-1, a translational regulator and stress granule-associated protein, to facilitate virus assembly. J. Virol. 88, 4434–4450. 10.1128/jvi.02607-13 24501406 PMC3993753

[B14] Barajas B. C. Tanaka M. Robinson B. A. Phuong D. J. Chutiraka K. Reed J. C.. (2018). Identifying the assembly intermediate in which Gag first associates with unspliced HIV-1 RNA suggests a novel model for HIV-1 RNA packaging. PLoS Pathog. 14:e1006977. 10.1371/journal.ppat.1006977 29664940 PMC5940231

[B15] Basu M. Courtney S. C. Brinton M. A. (2017). Arsenite-induced stress granule formation is inhibited by elevated levels of reduced glutathione in West Nile virus-infected cells. PLoS Pathog. 13:e1006240. 10.1371/journal.ppat.1006240 28241074 PMC5344523

[B16] Bechill J. Chen Z. Brewer J. W. Baker S. C. (2008). Coronavirus infection modulates the unfolded protein response and mediates sustained translational repression. J. Virol. 82, 4492–4501. 10.1128/jvi.00017-08 18305036 PMC2293058

[B17] Berezhna S. Y. Supekova L. Sever M. J. Schultz P. G. Deniz A. A. (2011). Dual regulation of hepatitis C viral RNA by cellular RNAi requires partitioning of Ago2 to lipid droplets and P-bodies. RNA 17, 1831–1845. 10.1261/rna.2523911 21868483 PMC3185916

[B18] Bhowmick R. Mukherjee A. Patra U. Chawla-Sarkar M. (2015). Rotavirus disrupts cytoplasmic P bodies during infection. Virus Res. 210, 344–354. 10.1016/j.virusres.2015.09.001 26386333

[B19] Bicknell A. A. Ricci E. P. (2017). When mRNA translation meets decay. Biochem. Soc. Trans. 45, 339–351. 10.1042/bst20160243 28408474

[B20] Bidet K. Dadlani D. Garcia-Blanco M. A. (2014). G3BP1, G3BP2 and CAPRIN1 are required for translation of interferon stimulated mRNAs and are targeted by a dengue virus non-coding RNA. PLoS Pathog. 10:e1004242. 10.1371/journal.ppat.1004242 24992036 PMC4081823

[B21] Bonenfant G. Williams N. Netzband R. Schwarz M. C. Evans M. J. Pager C. T. (2019). Zika virus subverts stress granules to promote and restrict viral gene expression. J. Virol. 93:e00520–19. 10.1128/JVI.00520-19 30944179 PMC6613768

[B22] Bordeleau M.-E. Matthews J. Wojnar J. M. Lindqvist L. Novac O. Jankowsky E.. (2005). Stimulation of mammalian translation initiation factor eIF4A activity by a small molecule inhibitor of eukaryotic translation. Proc. Natl.Acad. Sci. U.S.A. 102, 10460–10465. 10.1073/pnas.0504249102 16030146 PMC1176247

[B23] Bordeleau M. E. Mori A. Oberer M. Lindqvist L. Chard L. S. Higa T.. (2006). Functional characterization of IRESes by an inhibitor of the RNA helicase eIF4A. Nat. Chem. Biol. 2, 213–220. 10.1038/nchembio776 16532013

[B24] Borghese F. Michiels T. (2011). The leader protein of cardioviruses inhibits stress granule assembly. J. Virol. 85, 9614–9622. 10.1128/jvi.00480-11 21752908 PMC3165746

[B25] Bouttier M. Saumet A. Peter M. Courgnaud V. Schmidt U. Cazevieille C.. (2012). Retroviral GAG proteins recruit AGO2 on viral RNAs without affecting RNA accumulation and translation. Nucleic Acids Res. 40, 775–786. 10.1093/nar/gkr762 21948796 PMC3258151

[B26] Boyce M. Bryant K. F. Jousse C. Long K. Harding H. P. Scheuner D. (2005). A selective inhibitor of elF2α dephosphorylation protects cells from ER stress. Science 307, 935–939. 10.1126/science.1101902 15705855

[B27] Brocard M. Iadevaia V. Klein P. Hall B. Lewis G. Lu J. (2018). Norovirus infection results in assembly of virus-specific G3BP1 granules and evasion of eIF2α signaling. BioRxiv [Preprint]. 10.1101/490318

[B28] Burdick R. Smith J. L. Chaipan C. Friew Y. Chen J. Venkatachari N. J.. (2010). P body-associated protein Mov10 inhibits HIV-1 replication at multiple stages. J. Virol. 84, 10241–10253. 10.1128/jvi.00585-10 20668078 PMC2937795

[B29] Burgess H. M. Mohr I. (2018). Defining the role of stress granules in innate immune suppression by the herpes simplex virus 1 endoribonuclease VHS. J. Virol. 92, 1–15. 10.1128/jvi.00829-18 29793959 PMC6052315

[B30] Carroll K. Hastings C. Miller C. (2014). Amino acids 78 and 79 of mammalian orthoreovirus protein μNS are necessary for stress granule localization, core protein λ2 interaction, and *de novo* virus replication. Virology 71, 3831–3840. 10.1158/0008-5472.CAN-10-4002.BONE PMC3857589 24314644

[B31] Chable-Bessia C. Meziane O. Latreille D. Triboulet R. Zamborlini A. Wagschal A.. (2009). Suppression of HIV-1 replication by microRNA effectors. Retrovirology 6:26. 10.1186/1742-4690-6-26 19272132 PMC2657893

[B32] Chahar H. S. Chen S. Manjunath N. (2013). P-body components LSM1, GW182, DDX3, DDX6 and XRN1 are recruited to WNV replication sites and positively regulate viral replication. Virology 436, 1–7. 10.1016/j.virol.2012.09.041 23102969 PMC3545066

[B33] Chang H. W. Watson J. C. Jacobs B. L. (1992). The E3L gene of vaccinia virus encodes an inhibitor of the interferon-induced, double-stranded RNA-dependent protein kinase. Proc. Natl. Acad. Sci. U.S.A. 89, 4825–4829. 10.1073/pnas.89.11.4825 1350676 PMC49180

[B34] Chen C. Ma X. Hu Q. Li X. Huang F. Zhang J.. (2017). Moloney leukemia virus 10 (MOV10) inhibits the degradation of APOBEC3G through interference with the Vif-mediated ubiquitin-proteasome pathway. Retrovirology 14, 1–19. 10.1186/s12977-017-0382-1 29258557 PMC5735797

[B35] Chen D. Wilkinson C. R. M. Watt S. Penkett C. J. Toone W. M. Jones N.. (2008). Multiple pathways differentially regulate global oxidative stress responses in fission yeast dongrong. Mol. Biol. Cell. 19, 308–317. 10.1091/mbc.E07 18003976 PMC2174203

[B36] Choudhury P. Bussiere L. D. Miller L. (2017). Mammalian orthoreovirus factories modulate stress granule protein localization by interaction with G3BP1 promisree. J. Virol. 91, 1–23. 10.1128/JVI.01298-17 28794026 PMC5640875

[B37] Cimica V. Dalrymple N. A. Roth E. Nasonov A. Mackow E. R. (2014). An innate immunity-regulating virulence determinant is uniquely encoded within the andes virus nucleocapsid protein. MBio 5:e01088-135. 10.1128/mBio.01088-13 24549848 PMC3944819

[B38] Cinti A. Le Sage V. Ghanem M. Mouland A. J. (2016). HIV-1 gag blocks selenite-induced stress granule assembly by altering the mRNA cap-binding complex. MBio 7, 1–9. 10.1128/mbio.00329-16 27025252 PMC4817256

[B39] Cobos Jiménez V. Martinez F. O. Booiman T. van Dort K. A. van de Klundert M. A. A. Gordon S.. (2015). G3BP1 restricts HIV-1 replication in macrophages and T-cells by sequestering viral RNA. Virology 486, 94–104. 10.1016/j.virol.2015.09.007 26432022

[B40] Corcoran J. A. Johnston B. P. McCormick C. (2015). Viral Activation of MK2-hsp27-p115RhoGEF-RhoA signaling axis causes cytoskeletal rearrangements, P-body disruption and ARE-mRNA stabilization. PLoS Pathog. 11:e1004597. 10.1371/journal.ppat.1004597 25569678 PMC4287613

[B41] Corcoran J. A. Khaperskyy D. A. Johnston B. P. King C. A. Cyr D. P. Olsthoorn A. V.. (2012). Kaposi's sarcoma-associated herpesvirus G-protein-coupled receptor prevents AU-rich-element-mediated mRNA decay. J. Virol. 86, 8859–8871. 10.1128/JVI.00597-12 22696654 PMC3421767

[B42] Cougot N. Cavalier A. Thomas D. Gillet R. (2012). The dual organization of P-bodies revealed by immunoelectron microscopy and electron tomography. J. Mol. Biol. 420, 17–28. 10.1016/j.jmb.2012.03.027 22484175

[B43] Courtney S. C. Scherbik S. V. Stockman B. M. Brinton M. A. (2012). West Nile virus infections suppress early viral RNA synthesis and avoid inducing the cell stress granule response. J. Virol. 86, 3647–3657. 10.1128/jvi.06549-11 22258263 PMC3302502

[B44] Cristea I. M. Rozjabek H. Molloy K. R. Karki S. White L. L. Rice C. M.. (2010). Host factors associated with the sindbis virus RNA-dependent RNA polymerase: role for G3BP1 and G3BP2 in virus replication. J. Virol. 84, 6720–6732. 10.1128/jvi.01983-09 20392851 PMC2903289

[B45] Cuevas R. A. Ghosh A. Wallerath C. Hornung V. Coyne C. B. Sarkar S. N. (2016). MOV10 provides antiviral activity against RNA viruses by enhancing RIG-I–MAVS-independent IFN induction. J. Immunol. 196, 3877–3886. 10.4049/jimmunol.1501359 27016603 PMC4868630

[B46] Dauber B. Pelletier J. Smiley J. R. (2011). The herpes simplex virus 1 vhs protein enhances translation of viral true late mRNAs and virus production in a cell type-dependent manner. J. Virol. 85, 5363–5373. 10.1128/jvi.00115-11 21430045 PMC3094992

[B47] Dauber B. Poon D. dos Santos T. Duguay B. A. Mehta N. Saffran H. A.. (2016). The herpes simplex virus virion host shutoff protein enhances translation of viral true late mRNAs independently of suppressing protein kinase R and stress granule formation. J. Virol. 90, 6049–6057. 10.1128/jvi.03180-15 27099317 PMC4907241

[B48] Dauber B. Smiley J. R. Hay T. J. M. Finnen R. L. Banfield B. W. (2014). The herpes simplex virus 2 virion-associated ribonuclease vhs interferes with stress granule formation. J. Virol. 88, 12727–12739. 10.1128/jvi.01554-14 25142597 PMC4248931

[B49] Dhillon P. Namsa N. D. Sahoo L. Chorghade S. G. Tandra V. N. Rao C. D. (2018). Cytoplasmic relocalization and colocalization with viroplasms of host cell proteins, and their role in rotavirus infection. J. Virol. 92, 1–24. 10.1128/jvi.00612-18 29769336 PMC6052293

[B50] Dhillon P. Rao C. D. (2018). Rotavirus induces formation of remodeled stress granules and P bodies and their sequestration in viroplasms to promote progeny virus production. J. Virol. 92, 1–26. 10.1128/jvi.01363-18 30258011 PMC6258942

[B51] Dickey L. L. Duncan J. K. Hanley T. M. Fearns R. (2015). Decapping protein 1 phosphorylation modulates IL-8 expression during respiratory syncytial virus infection. Virology 481, 199–209. 10.1016/j.virol.2015.02.043 25796077 PMC4437874

[B52] Dimasi P. Quintiero A. Shelkovnikova T. A. Buchman V. L. (2017). Modulation of p-eIF2α cellular levels and stress granule assembly/disassembly by trehalose. Sci. Rep. 7:44088. 10.1038/srep44088 28276506 PMC5343430

[B53] Dinh P. X. Beura L. K. Das P. B. Panda D. Das A. Pattnaik A. K. (2012). Induction of stress granule-like structures in vesicular stomatitis virus-infected cells. J. Virol. 87, 372–383. 10.1128/jvi.02305-12 23077311 PMC3536414

[B54] Dixit U. Pandey A. K. Mishra P. Sengupta A. Pandey V. N. (2016). Staufen1 promotes HCV replication by inhibiting protein kinase R and transporting viral RNA to the site of translation and replication in the cells. Nucleic Acids Res. 44, 5271–5287. 10.1093/nar/gkw312 27106056 PMC4914112

[B55] Dobra I. Pankivskyi S. Samsonova A. Pastre D. Hamon L. (2018). Relation between stress granules and cytoplasmic protein aggregates linked to neurodegenerative diseases. Curr. Neurol. Neurosci. Rep. 18:107. 10.1007/s11910-018-0914-7 30406848

[B56] Dong Y. Yang J. Ye W. Wang Y. Miao Y. Ding T.. (2015). LSm1 binds to the dengue virus RNA 3′ UTR and is a positive regulator of dengue virus replication. Int. J. Mol. Med. 35, 1683–1689. 10.3892/ijmm.2015.2169 25872476

[B57] Dougherty J. D. Tsai W. C. Lloyd R. E. (2015). Multiple poliovirus proteins repress cytoplasmic RNA granules. Viruses 7, 6127–6140. 10.3390/v7122922 26610553 PMC4690851

[B58] Dougherty J. D. White J. P. Lloyd R. E. (2011). Poliovirus-mediated disruption of cytoplasmic processing bodies. J. Virol. 85, 64–75. 10.1128/jvi.01657-10 20962086 PMC3014174

[B59] Edgar R. C. (2004). MUSCLE: multiple sequence alignment with high accuracy and high throughput. Nucleic Acids Res. 32, 1792–1797. 10.1093/nar/gkh340 15034147 PMC390337

[B60] Emara M. M. Brinton M. A. (2007). Interaction of TIA-1/TIAR with West Nile and dengue virus products in infected cells interferes with stress granule formation and processing body assembly. Proc. Natl.Acad. Sci. U.S.A. 104, 9041–9046. 10.1073/pnas.0703348104 17502609 PMC1885624

[B61] Esclatine A. Taddeo B. Roizman B. (2004). Herpes simplex virus 1 induces cytoplasmic accumulation of TIA-1/TIAR and both synthesis and cytoplasmic accumulation of tristetraprolin, two cellular proteins that bind and destabilize AU-rich RNAs. J. Virol. 78, 8582–8592. 10.1128/JVI.78.16.8582 15280467 PMC479066

[B62] Farny N. G. Kedersha N. L. Silver P. A. (2009). Metazoan stress granule assembly is mediated by P-eIF2α-dependent and -independent mechanisms. RNA 15, 1814–1821. 10.1261/rna.1684009 19661161 PMC2743051

[B63] Fay M. M. Anderson P. J. (2018). The role of RNA in biological phase separations. J. Mol. Biol. 430, 4685–4701. 10.1016/j.jmb.2018.05.003 29753780 PMC6204303

[B64] Fields B. N. Knipe D. M. Howley P. M. (2013). Fields Virology. Philadelphia: Wolters Kluwer Health/Lippincott Williams & Wilkins.

[B65] Finnen R. L. Pangka K. R. Banfield B. W. (2012). Herpes simplex virus 2 infection impacts stress granule accumulation. J. Virol. 86, 8119–8130. 10.1128/jvi.00313-12 22623775 PMC3421649

[B66] Finnen R. L. Zhu M. Li J. Romo D. Banfield B. W. (2016). Herpes simplex virus 2 virion host shutoff endoribonuclease activity is required to disrupt stress granule formation. J. Virol. 90, 7943–7955. 10.1128/JVI.00947-16 27334584 PMC4988144

[B67] Fiorini F. Robin J.-P. Kanaan J. Borowiak M. Croquette V. Le Hir H.. (2018). HTLV-1 Tax plugs and freezes UPF1 helicase leading to nonsense-mediated mRNA decay inhibition. Nat. Commun. 9:431. 10.1038/s41467-017-02793-6 29382845 PMC5789848

[B68] Fontaine K. A. Leon K. E. Khalid M. M. Tomar S. Jimenez-Morales D. Dunlap M.. (2018). The cellular NMD pathway restricts zika virus infection and is targeted by the viral capsid protein. MBio 9, 1–12. 10.1128/mBio.02126-18 30401782 PMC6222128

[B69] Fournier M. J. Gareau C. Mazroui R. (2010). The chemotherapeutic agent bortezomib induces the formation of stress granules. Cancer Cell Int. 10, 1–10. 10.1186/1475-2867-10-12 20429927 PMC2873330

[B70] Fricke J. Koo L. Y. Brown C. R. Collins P. L. (2012). p38 and OGT sequestration into viral inclusion bodies in cells infected with human respiratory syncytial virus suppresses MK2 activities and stress granule assembly. J. Virol. 87, 1333–1347. 10.1128/jvi.02263-12 23152511 PMC3554139

[B71] Friedman E. Karen K. A. (2017). Effects of adenovirus infection on the localization of cellular protein pat1b effects of adenovirus infection on the localization of cellular protein. Georg. J. Sci. 75:15.

[B72] Fritzlar S. Aktepe T. Chao Y. W. McAllaster M. Wilen C. White P.. (2019). Mouse norovirus infection arrests host cell translation uncoupled from the stress granule-PKR-eIF2α axis. mBio 10, e00960–19. 10.1128/mBio.00960-19 31213553 PMC6581855

[B73] Frolova E. Gorchakov R. Garmashova N. Atasheva S. Vergara L. A. Frolov I. (2006). Formation of nsP3-specific protein complexes during sindbis virus replication. J. Virol. 80, 4122–4134. 10.1128/jvi.80.8.4122-4134.2006 16571828 PMC1440443

[B74] Fros J. J. Domeradzka N. E. Baggen J. Geertsema C. Flipse J. Vlak J. M.. (2012). Chikungunya virus nsP3 blocks stress granule assembly by recruitment of G3BP into cytoplasmic foci. J. Virol. 86, 10873–10879. 10.1128/jvi.01506-12 22837213 PMC3457282

[B75] Fujimura K. Sasaki A. T. Anderson P. (2012). Selenite targets eIF4E-binding protein-1 to inhibit translation initiation and induce the assembly of non-canonical stress granules. Nucleic Acids Res. 40, 8099–8110. 10.1093/nar/gks566 22718973 PMC3439927

[B76] Fung G. Ng C. S. Zhang J. Shi J. Wong J. Piesik P.. (2013). Production of a dominant-negative fragment due to G3BP1 cleavage contributes to the disruption of mitochondria-associated protective stress granules during CVB3 infection. PLoS ONE 8:e79546. 10.1371/journal.pone.0079546 24260247 PMC3832613

[B77] Galan A. Lozano G. Piñeiro D. Martinez-Salas E. (2017). G3BP1 interacts directly with the FMDV IRES and negatively regulates translation. FEBS J. 284, 3202–3217. 10.1111/febs.14184 28755480

[B78] Garaigorta U. Heim M. H. Boyd B. Wieland S. Chisari F. V. (2012). Hepatitis C virus (HCV) induces formation of stress granules whose proteins regulate HCV RNA replication and virus assembly and egress. J. Virol. 86, 11043–11056. 10.1128/jvi.07101-11 22855484 PMC3457181

[B79] Garcez P. Nascimento J. Mota de Vasconcelos J. Madeiro da Costa R. Delvecchio R. Trindade P.. (2017). Zika virus disrupts molecular fingerprinting of human neurospheres. Sci. Rep. 7, 1–10. 10.1038/srep40780 28112162 PMC5256095

[B80] Gaucherand L. Porter B. K. Levene R. E. Price E. L. Schmaling S. K. Rycroft C. H.. (2019). The influenza A virus endoribonuclease PA-X usurps host mRNA processing machinery to limit host gene expression. Cell Rep. 27, 776–792.e7. 10.1016/j.celrep.2019.03.063 30995476 PMC6499400

[B81] Glaunsinger B. Ganem D. (2004). Lytic KSHV infection inhibits host gene expression by accelerating global mRNA turnover. Mol. Cell. 13, 713–723. 10.1016/S1097-2765(04)00091-7 15023341

[B82] Gorchakov R. Garmashova N. Frolova E. Frolov I. (2008). Different types of nsP3-containing protein complexes in sindbis virus-infected cells. J. Virol. 82, 10088–10101. 10.1128/jvi.01011-08 18684830 PMC2566286

[B83] Greer A. E. Hearing P. Ketner G. (2011). The adenovirus E4 11k protein binds and relocalizes the cytoplasmic P-body component Ddx6 to aggresomes. Virology 417, 161–168. 10.1016/j.virol.2011.05.017 21700307 PMC3152696

[B84] Groskreutz D. J. Babor E. C. Monick M. M. Varga S. M. Hunninghake G. W. (2010). Respiratory syncytial virus limits α subunit of eukaryotic translation initiation factor 2 (eIF2α) phosphorylation to maintain translation and viral replication. J. Biol. Chem. 285, 24023–24031. 10.1074/jbc.m109.077321 20519500 PMC2911276

[B85] Guzikowski A. R. Chen Y. S. Zid B. M. (2019). Stress-induced mRNP granules: form and function of processing bodies and stress granules. Wiley Interdiscip. Rev. RNA 10:e1524. 10.1002/wrna.1524 30793528 PMC6500494

[B86] Habjan M. Pichlmair A. Elliott R. M. Överby A. K. Glatter T. Gstaiger M.. (2009). NSs protein of rift valley fever virus induces the specific degradation of the double-stranded RNA-dependent protein kinase. J. Virol. 83, 4365–4375. 10.1128/JVI.02148-08 19211744 PMC2668506

[B87] Han A.-P. Yu C. Lu L. Fujiwara Y. Browne C. Chin G.. (2001). Heme-regulated eIF2α kinase (HRI) is required for translational regulation and survival of erythroid precursors in iron deficiency. EMBO J. 20, 6909–6918. 10.1093/emboj/20.23.6909 11726526 PMC125753

[B88] Hanley L. L. Teng M. N. Fearns R. Collins P. L. Djang R. McGivern D. R. (2010). Roles of the respiratory syncytial virus trailer region: effects of mutations on genome production and stress granule formation. Virology 406, 241–252. 10.1016/j.virol.2010.07.006 20701943 PMC2971657

[B89] Harding H. P. Zhang Y. Bertolotti A. Zeng H. Ron D. (2000). Perk is essential for translational regulation and cell survival during the unfolded protein response. Mol. Cell. 5, 897–904. 10.1016/S1097-2765(00)80330-5 10882126

[B90] Hashimoto S. Yamamoto S. Ogasawara N. Sato T. Yamamoto K. Katoh H.. (2016). Mumps virus induces protein-kinase-R-dependent stress granules, partly suppressing type III interferon production. PLoS ONE 11:e0161793. 10.1371/journal.pone.0161793 27560627 PMC4999214

[B91] Hopkins K. C. Tartell M. A. Herrmann C. Hackett B. A. Taschuk F. Panda D.. (2015). Virus-induced translational arrest through 4EBP1/2-dependent decay of 5′-TOP mRNAs restricts viral infection. Proc. Natl.Acad. Sci. U.S.A. 112, E2920–E2929. 10.1073/pnas.1418805112 26038567 PMC4460451

[B92] Hou S. Kumar A. Xu Z. Airo A. M. Stryapunina I. Wong C. P.. (2017). Zika virus hijacks stress granule proteins and modulates the host stress response. J. Virol. 91, 1–21. 10.1128/JVI.00474-17 28592527 PMC5533921

[B93] Hu Z. Wang Y. Tang Q. Yang X. Qin Y. Chen M. (2018). Inclusion bodies of human parainfluenza virus type 3 inhibit antiviral stress granule formation by shielding viral RNAs. PLoS Pathog. 14:e1006948. 10.1371/journal.ppat.1006948 29518158 PMC5860793

[B94] Huang C. Lokugamage K. G. Rozovics J. M. Narayanan K. Semler B. L. Makino S. (2011). SARS coronavirus nsp1 protein induces template-dependent endonucleolytic cleavage of mRNAs: Viral mRNAs are resistant to nsp1-induced RNA cleavage. PLoS Pathog. 7:e1002433. 10.1371/journal.ppat.1002433 22174690 PMC3234236

[B95] Hubstenberger A. Courel M. Bénard M. Souquere S. Ernoult-Lange M. Chouaib R.. (2017). P-body purification reveals the condensation of repressed mRNA regulons. Mol. Cell. 68, 144–157.e5. 10.1016/j.molcel.2017.09.003 28965817

[B96] Humoud M. N. Doyle N. Royall E. Willcocks M. M. Sorgeloos F. van Kuppeveld F.. (2016). Feline calicivirus infection disrupts assembly of cytoplasmic stress granules and induces G3BP1 cleavage. J. Virol. 90, 6489–6501. 10.1128/jvi.00647-16 27147742 PMC4936126

[B97] Ikegami T. Narayanan K. Won S. Kamitani W. Peters C. J. Makino S. (2009). Rift valley fever virus NSs protein promotes post-transcriptional downregulation of protein kinase PKR and inhibits eIF2α phosphorylation. PLOS Pathog. 5:e1000287. 10.1371/journal.ppat.1000287 19197350 PMC2629125

[B98] Iseni F. Garcin D. Nishio M. Kedersha N. Anderson P. Kolakofsky D. (2002). Sendai virus trailer RNA binds TIAR, a cellular protein involved in virus-induced apoptosis. EMBO J. 21, 5141–5150. 10.1093/emboj/cdf513 12356730 PMC129035

[B99] Isler J. A. Maguire T. G. Alwine J. C. (2005a). Production of infectious human cytomegalovirus virions is inhibited by drugs that disrupt calcium homeostasis in the endoplasmic reticulum. J. Virol. 79, 15388–15397. 10.1128/jvi.79.24.15388-15397.2005 16306610 PMC1316032

[B100] Isler J. A. Skalet A. H. Alwine J. C. (2005b). Human cytomegalovirus infection activates and regulates the unfolded protein response. J. Virol. 79, 6890–6899. 10.1128/jvi.79.11.6890-6899.2005 15890928 PMC1112127

[B101] Jaaskelainen K. Kaukinen P. Minskaya E. S. Plyusnina A. Vapalahti O. Elliott R. M. (2009). Tula and puumala hantavirus NSs ORFs are functional and the products inhibit activation of the interferon-beta promoter. J. Med. Virol. 1304, 1298–1304. 10.1002/jmv 17705180

[B102] Jackrel M. E. Desantis M. E. Martinez B. A. Castellano L. M. Stewart R. M. Caldwell K. A.. (2014). Potentiated Hsp104 variants antagonize diverse proteotoxic misfolding events. Cell 156, 170–182. 10.1016/j.cell.2013.11.047 24439375 PMC3909490

[B103] Jain S. Wheeler J. R. Walters R. Agrawal R. W. Barsic A. Parker R. (2016). ATPase modulated stress granules contain a diverse proteome and substructure Saumya. Cell 164, 487–498. 10.1016/j.cell.2015.12.038 26777405 PMC4733397

[B104] Jangra R. K. Yi M. Lemon S. M. (2010). DDX6 (Rck/p54) is required for efficient hepatitis C virus replication but not for internal ribosome entry site-directed translation. J. Virol. 84, 6810–6824. 10.1128/JVI.00397-10 20392846 PMC2903299

[B105] Jefferson M. Donaszi-Ivanov A. Pollen S. Dalmay T. Saalbach G. Powell P. P. (2014). Host factors that interact with the pestivirus N-terminal protease, Npro, are components of the ribonucleoprotein complex. J. Virol. 88, 10340–10353. 10.1128/jvi.00984-14 24965446 PMC4178888

[B106] Jiang H.-Y. Wek R. C. (2005). GCN2 phosphorylation of eIF2alpha activates NF-kappaB in response to UV irradiation. Biochem. J. 385, 371–80. 10.1042/BJ20041164 15355306 PMC1134707

[B107] Kamitani W. Huang C. Narayanan K. Lokugamage K. G. Makino S. (2009). A two-pronged strategy to suppress host protein synthesis by SARS coronavirus Nsp1 protein. Nat. Struct. Mol. Biol. 16, 1134–1140. 10.1038/nsmb.1680 19838190 PMC2784181

[B108] Katoh H. Okamoto T. Fukuhara T. Kambara H. Morita E. Mori Y.. (2013). Japanese encephalitis virus core protein inhibits stress granule formation through an interaction with caprin-1 and facilitates viral propagation. J. Virol. 87, 489–502. 10.1128/jvi.02186-12 23097442 PMC3536427

[B109] Katsafanas G. C. Moss B. (2004). Vaccinia virus intermediate stage transcription is complemented by Ras-GTPase-activating protein SH3 domain-binding protein (G3BP) and cytoplasmic activation/proliferation-associated protein (p137) individually or as a heterodimer. J. Biol. Chem. 279, 52210–52217. 10.1074/jbc.M411033200 15471883

[B110] Katsafanas G. C. Moss B. (2007). Linkage of transcription and translation within cytoplasmic poxvirus DNA factories provides a mechanism to coordinat viral and usurop functions. Cell Host Microbe 2, 221–228. 10.1016/j.chom.2007.08.005 18005740 PMC2084088

[B111] Kedersha N. Anderson P. Liu J. O. Romo D. Kaufman R. Gorospe M.. (2006). Eukaryotic initiation factor 2α-independent pathway of stress granule induction by the natural product pateamine A. J. Biol. Chem. 281, 32870–32878. 10.1074/jbc.m606149200 16951406

[B112] Kedersha N. Gupta M. Li W. Miller I. Anderson P. (1999). RNA-binding Proteins TIA-1 and TIAR Link the Phosphorylation of eIF-2a to the Assembly of Mammalian Stress Granules. J. Cell Biol. 147, 1431–1441. 10613902 10.1083/jcb.147.7.1431 PMC2174242

[B113] Kedersha N. Ivanov P. Anderson P. (2011). Stress granules and cell signaling: more than just a passing phase? Trends Biochem. Sci. 4, 1–23. 10.1126/scisignal.2001449 PMC3832949 24029419

[B114] Kedersha N. Stoecklin G. Ayodele M. Yacono P. Lykke-Andersen J. Fitzler M. J.. (2005). Stress granules and processing bodies are dynamically linked sites of mRNP remodeling. J. Cell Biol. 169, 871–884. 10.1083/jcb.200502088 15967811 PMC2171635

[B115] Khaperskyy D. A. Emara M. M. Johnston B. P. Anderson P. Hatchette T. F. McCormick C. (2014). Influenza A virus host shutoff disables antiviral stress-induced translation arrest. PLoS Pathog. 10:e1004217. 10.1371/journal.ppat.1004217 25010204 PMC4092144

[B116] Khaperskyy D. A. Hatchette T. F. McCormick C. (2011). Influenza A virus inhibits cytoplasmic stress granule formation. FASEB J. 26, 1629–1639. 10.1096/fj.11-196915 22202676

[B117] Khong A. Jan E. (2011). Modulation of stress granules and P bodies during dicistrovirus infection. J. Virol. 85, 1439–1451. 10.1128/jvi.02220-10 21106737 PMC3028890

[B118] Khong A. Kerr C. H. Yeung C. H. L. Keatings K. Nayak A. Allan D. W.. (2016). Disruption of stress granule formation by the multifunctional cricket paralysis virus 1A protein. J. Virol. 91, 1–20. 10.1128/jvi.01779-16 28003491 PMC5309961

[B119] Kim D. Y. Reynaud J. M. Rasalouskaya A. Akhrymuk I. Mobley J. A. Frolov I.. (2016). New world and old world alphaviruses have evolved to exploit different components of stress granules, FXR and G3BP Proteins, for assembly of viral replication complexes. PLoS Pathog. 12:e1005810. 10.1371/journal.ppat.1005810 27509095 PMC4980055

[B120] Kimball S. R. Horetsky R. L. Ron D. Jefferson L. S. Harding P. Harding H. P.. (2002). Mammalian stress granules represent sites of accumulation of stalled translation initiation complexes. Am. J. Physiol. Cell Physiol. 284, C273–284. 10.1152/ajpcell.00314.2002 12388085

[B121] King B. R. Hershkowitz D. Eisenhauer P. L. Weir M. E. Ziegler C. M. Russo J.. (2017). A map of the arenavirus nucleoprotein-host protein interactome reveals that junín virus selectively impairs the antiviral activity of double-stranded RNA-activated protein kinase (PKR). J. Virol. 91, 1–24. 10.1128/jvi.00763-17 28539447 PMC5512243

[B122] Kint J. Langereis M. A. Maier H. J. Britton P. van Kuppeveld F. J. Koumans J.. (2016). Infectious bronchitis coronavirus limits interferon production by inducing a host shutoff that requires accessory protein 5b. J. Virol. 90, 7519–7528. 10.1128/jvi.00627-16 27279618 PMC4984617

[B123] Kohl A. Clayton R. F. Weber F. Bridgen A. Randall R. E. Elliott R. M. (2003). Bunyamwera virus nonstructural protein NSs counteracts interferon regulatory factor 3-mediated induction of early cell death. J. Virol. 77, 7999–8008. 10.1128/JVI.77.14.7999-8008.2003 12829839 PMC161919

[B124] Krahling V. Stein D. A. Spiegel M. Weber F. Muhlberger E. (2008). Severe acute respiratory syndrome coronavirus triggers apoptosis via protein kinase R but is resistant to its antiviral activity. J. Virol. 83, 2298–2309. 10.1128/jvi.01245-08 19109397 PMC2643707

[B125] Kroschwald S. Maharana S. Simon A. (2017). Hexanediol: a chemical probe to investigate the material properties of membrane-less compartments. Matters 1–7. 10.19185/matters.201702000010

[B126] Langereis M. A. Feng Q. van Kuppeveld F. J. (2013). MDA5 localizes to stress granules, but this localization is not required for the induction of type I interferon. J. Virol. 87, 6314–6325. 10.1128/JVI.03213-12 23536668 PMC3648107

[B127] Lawrence P. Schafer E. A. Rieder E. (2012). The nuclear protein Sam68 is cleaved by the FMDV 3C protease redistributing Sam68 to the cytoplasm during FMDV infection of host cells. Virology 425, 40–52. 10.1016/j.virol.2011.12.019 22280896

[B128] Le Sage V. Cinti A. McCarthy S. Amorim R. Rao S. Daino G. L.. (2016). Ebola virus VP35 blocks stress granule assembly. Virology 502, 73–83. 10.1016/j.virol.2016.12.012 28013103

[B129] Legros S. Boxus M. Gatot J. S. Van Lint C. Kruys V. Kettmann R.. (2011). The HTLV-1 Tax protein inhibits formation of stress granules by interacting with histone deacetylase 6. Oncogene 30, 4050–4062. 10.1038/onc.2011.120 21532619

[B130] Li Q. Pène V. Krishnamurthy S. Cha H. Liang T. J. (2013). Hepatitis C virus infection activates an innate pathway involving IKK-α in lipogenesis and viral assembly. Nat. Med. 19, 722–729. 10.1038/nm.3190 23708292 PMC3676727

[B131] Li T. Li X. Zhu W. F. Wang H. Y. Mei L. Wu S. Q.. (2016). NF90 is a novel influenza A virus NS1-interacting protein that antagonizes the inhibitory role of NS1 on PKR phosphorylation. FEBS Lett. 590, 2797–2810. 10.1002/1873-3468.12311 27423063

[B132] Li W. Li Y. Kedersha N. Anderson P. Emara M. Swiderek K. M.. (2002). Cell proteins TIA-1 and TIAR interact with the 3' stem-loop of the West Nile virus complementary minus-strand RNA and facilitate virus replication. J. Virol. 76, 11989–12000. 10.1128/JVI.76.23.11989 12414941 PMC136884

[B133] Li Z. Ning S. Su X. Liu X. Wang H. Liu Y.. (2018). Enterovirus 71 antagonizes the inhibition of the host intrinsic antiviral factor A3G. Nucleic Acids Res. 46, 11514–11527. 10.1093/nar/gky840 30247716 PMC6265463

[B134] Li Z. Okonski K. M. Samuel C. E. (2012). Adenosine deaminase acting on RNA 1 (ADAR1) suppresses the induction of interferon by measles virus. J. Virol. 86, 3787–3794. 10.1128/jvi.06307-11 22278222 PMC3302501

[B135] Liao Y. Fung T. S. Huang M. Fang S. G. Zhong Y. Liu D. X. (2013). Upregulation of CHOP/GADD153 during coronavirus infectious bronchitis virus infection modulates apoptosis by restricting activation of the extracellular signal-regulated kinase pathway. J. Virol. 87, 8124–8134. 10.1128/jvi.00626-13 23678184 PMC3700216

[B136] Liem J. Liu J. (2016). Stress beyond translation: poxviruses and more. Viruses 8, 1–20. 10.3390/v8060169 27314378 PMC4926189

[B137] Lindquist M. E. Lifland A. W. Utley T. J. Santangelo P. J. Crowe J. E. (2010). Respiratory syncytial virus induces host RNA stress granules to facilitate viral replication. J. Virol. 84, 12274–12284. 10.1128/jvi.00260-10 20844027 PMC2976418

[B138] Lindquist M. E. Mainou B. A. Dermody T. S. Crowe J. E. (2011). Activation of protein kinase R is required for induction of stress granules by respiratory syncytial virus but dispensable for viral replication. Virology 413, 103–110. 10.1016/j.virol.2011.02.009 21377708 PMC3072468

[B139] Linero F. N. Thomas M. G. Boccaccio G. L. Scolaro L. A. (2011). Junín virus infection impairs stress-granule formation in Vero cells treated with arsenite via inhibition of eiF2α phosphorylation. J. Gen. Virol. 92, 2889–2899. 10.1099/vir.0.033407-0 21813702

[B140] Lingappa J. R. Hill R. L. Wong M. L. Hegde R. S. (1997). A multistep, ATP-dependent pathway for assembly of human immunodeficiency virus capsids in a cell-free system. J. Cell Biol. 136, 567–581. 10.1083/jcb.136.3.567 9024688 PMC2134302

[B141] Liu J. McFadden G. (2014). SAMD9 Is an innate antiviral host factor with stress response properties that can be antagonized by poxviruses. J. Virol. 89, 1925–1931. 10.1128/jvi.02262-14 25428864 PMC4300762

[B142] Loureiro M. E. Zorzetto-Fernandes A. L. Radoshitzky S. Chi X. Dallari S. Marooki N.. (2018). DDX3 suppresses type I interferons and favors viral replication during Arenavirus infection. PLoS Pathog. 14:e1007125. 10.1371/journal.ppat.1007125 30001425 PMC6042795

[B143] Luo Y. Na Z. Slavoff S. A. (2018). P-Bodies: composition, properties, and functions. Biochemistry 57, 2424–2431. 10.1021/acs.biochem.7b01162 29381060 PMC6296482

[B144] Mahboubi H. Stochaj U. (2017). Cytoplasmic stress granules: dynamic modulators of cell signaling and disease. Biochim. Biophys. Acta 1863, 884–895. 10.1016/j.bbadis.2016.12.022 28095315

[B145] Manners O. Baquero-Perez B. Whitehouse A. (2018). m6A: widespread regulatory control in virus replication. Biochim. Biophys. Acta 1862, 370–381. 10.1016/j.bbagrm.2018.10.015 30412798 PMC6414752

[B146] Marshall E. E. Bierle C. J. Brune W. Geballe A. P. (2009). Essential role for either TRS1 or IRS1 in human cytomegalovirus replication. J. Virol. 83, 4112–4120. 10.1128/jvi.02489-08 19211736 PMC2668495

[B147] Martin K. L. Johnson M. D'Aquila R. T. (2011). APOBEC3G complexes decrease human immunodeficiency virus type 1 production. J. Virol. 86, 8916–8916. 10.1128/jvi.01199-12 PMC3165773 21752914

[B148] Mateer E. J. Paessler S. Huang C. (2018). Visualization of double-stranded RNA colocalizing with pattern recognition receptors in arenavirus infected cells. Front. Cell. Infect. Microbiol. 8:251. 10.3389/fcimb.2018.00251 30087859 PMC6066581

[B149] Matthews J. D. Frey T. K. (2012). Analysis of subcellular G3BP redistribution during rubella virus infection. J. Gen. Virol. 93, 267–274. 10.1099/vir.0.036780-0 21994324 PMC3352340

[B150] Matthys V. S. Cimica V. Dalrymple N. A. Glennon N. B. Bianco C. Mackow E. R. (2014). Hantavirus GnT elements mediate TRAF3 binding and inhibit RIG-I/TBK1-directed beta interferon transcription by blocking IRF3 phosphorylation. J. Virol. 88, 2246–2259. 10.1128/JVI.02647-13 24390324 PMC3911538

[B151] Mazroui R. Di Marco S. Kaufman R. J. Gallouzi I. E. (2007). Inhibition of the ubiquitin-proteasome system induces stress granule formation. Mol. Biol. Cell. 18, 986–994. 10.1091/mbc.E06-12-1079 17475769 PMC1924830

[B152] Mazroui R. Sukarieh R. Bordeleau M. Kaufman R. Northcote P. Tanaka J. (2006). Inhibition of ribosome recruitment induces stress granule formation independently of eukaryotic initiation factor 2 phosphorylation. Mol. Biol. Cell. 17, 4212–4219. 10.1091/mbc.e06-04-0318 16870703 PMC1635342

[B153] McEwen E. Kedersha N. Song B. Scheuner D. Gilks N. Han A.. (2005). Heme-regulated inhibitor kinase-mediated phosphorylation of eukaryotic translation initiation factor 2 inhibits translation, induces stress granule formation, and mediates survival upon arsenite exposure. J. Biol. Chem. 280, 16925–16933. 10.1074/jbc.M412882200 15684421

[B154] McInerney G. M. Kedersha N. Kaufman R. J. Anderson P. Liljestrom P. (2005). Importance of eIF2a phosphorylation and stress granule assembly in alphavirus translation regulation. Mol. Biol. Cell. 16, 645–657. 10.1091/mbc.E05 PMC1182313 15930128

[B155] Meng X. Xiang Y. (2019). RNA granules associated with SAMD9-mediated poxvirus restriction are similar to antiviral granules in composition but do not require TIA1 for poxvirus restriction. Virology 529, 16–22. 10.1016/j.virol.2019.01.007 30641480 PMC6382536

[B156] Mir M. A. Duran W. A. Hjelle B. L. Ye C. Panganiban A. T. (2008). Storage of cellular 5' mRNA caps in P bodies for viral cap-snatching. Proc. Natl. Acad. Sci. U.S.A. 105, 19294–19299. 10.1073/pnas.0807211105 19047634 PMC2614755

[B157] Mocquet V. Neusiedler J. Rende F. Cluet D. Robin J.-P. Terme J.-M.. (2012). The human T-lymphotropic virus type 1 tax protein inhibits nonsense-mediated mRNA decay by interacting with INT6/EIF3E and UPF1. J. Virol. 86, 7530–7543. 10.1128/jvi.07021-11 22553336 PMC3416306

[B158] Mohankumar V. Dhanushkodi N. R. Raju R. (2011). Sindbis virus replication, is insensitive to rapamycin and torin1, and suppresses Akt/mTOR pathway late during infection in HEK cells. Biochem. Biophys. Res. Commun. 406, 262–267. 10.1016/j.bbrc.2011.02.030 21316343 PMC3073402

[B159] Mok B. W.-Y. Song W. Wang P. Tai H. Chen Y. Zheng M.. (2012). The NS1 protein of influenza A virus interacts with cellular processing bodies and stress granules through RNA-associated protein 55 (RAP55) during virus infection. J. Virol. 86, 12695–12707. 10.1128/jvi.00647-12 22973032 PMC3497642

[B160] Mollet S. Cougot N. Wilczynska A. Dautry F. Kress M. Bertrand E.. (2008). Translationally repressed mRNA transiently cycles through stress granules during stress. Mol. Biol. Cell. 19, 4469–79. 10.1091/mbc.E08 18632980 PMC2555929

[B161] Montero H. Rojas M. Arias C. F. Lopez S. (2008). Rotavirus infection induces the phosphorylation of eIF2 but prevents the formation of stress granules. J. Virol. 82, 1496–1504. 10.1128/jvi.01779-07 18032499 PMC2224440

[B162] Moon S. L. Anderson J. R. Kumagai Y. Wilusz C. J. Akira S. Khromykh A. A.. (2012). A noncoding RNA produced by arthropod-borne flaviviruses inhibits the cellular exoribonuclease XRN1 and alters host mRNA stability. RNA 18, 2029–2040. 10.1261/rna.034330.112 23006624 PMC3479393

[B163] Moon S. L. Blackinton J. G. Anderson J. R. Dozier M. K. Dodd B. J. T. Keene J. D.. (2015). XRN1 stalling in the 5' UTR of hepatitis C virus and bovine viral diarrhea virus is associated with dysregulated host mRNA stability. PLoS Pathog. 11:e1004708. 10.1371/journal.ppat.1004708 25747802 PMC4352041

[B164] Moon S. L. Morisaki T. Khong A. Lyon K. Parker R. Stasevich T. J. (2019). Multicolour single-molecule tracking of mRNA interactions with RNP granules. Nat. Cell Biol. 21, 162–168. 10.1038/s41556-018-0263-4 30664789 PMC6375083

[B165] Mulvey M. Poppers J. Sternberg D. Mohr I. (2003). Regulation of eIF2 phosphorylation by different functions that act during discrete phases in the herpes simplex virus type 1 life cycle. J. Virol. 77, 10917–10928. 10.1128/jvi.77.20.10917-10928.2003 14512542 PMC225003

[B166] Nakagawa K. Narayanan K. Wada M. Makino S. (2018). Inhibition of stress granule formation by middle east respiratory syndrome coronavirus 4a accessory protein facilitates viral translation, leading to efficient virus replication. J. Virol. 92, 1–19. 10.1128/jvi.00902-18 30068649 PMC6158436

[B167] Nakano K. Ando T. Yamagishi M. Yokoyama K. Ishida T. Ohsugi T.. (2013). Viral interference with host mRNA surveillance, the nonsense-mediated mRNA decay (NMD) pathway, through a new function of HTLV-1 Rex: implications for retroviral replication. Microbes Infect. 15, 491–505. 10.1016/j.micinf.2013.03.006 23541980

[B168] Narayanan K. Huang C. Lokugamage K. Kamitani W. Ikegami T. Tseng C.-T. K.. (2008). Severe acute respiratory syndrome coronavirus nsp1 suppresses host gene expression, including that of type i interferon, in infected cells. J. Virol. 82, 4471–4479. 10.1128/jvi.02472-07 18305050 PMC2293030

[B169] Nathans R. Chu C. ying Serquina A. K. Lu C. C. Cao H. Rana T. M. (2009). Cellular microRNA and P bodies modulate host-HIV-1 interactions. Mol. Cell 34, 696–709. 10.1016/j.molcel.2009.06.003 19560422 PMC2763548

[B170] Nelson E. V. Schmidt K. M. Dog S. Banadyga L. Olejnik J. Hume A. J. (2016). Ebola virus does not induce stress granule formation during infection and sequesters stress granule proteins within viral inclusions. J. Virol. 90, 7268–7284. 10.1128/JVI.00459-16 27252530 PMC4984654

[B171] Ng C. S. Jogi M. Yoo J.-S. Onomoto K. Koike S. Iwasaki T.. (2013). Encephalomyocarditis virus disrupts stress granules, the critical platform for triggering antiviral innate immune responses. J. Virol. 87, 9511–9522. 10.1128/jvi.03248-12 23785203 PMC3754122

[B172] Niewidok B. Igaev M. Pereira da Graca A. Strassner A. Lenzen C. Richter C. P.. (2018). Single-molecule imaging reveals dynamic biphasic partition of RNA-binding proteins in stress granules. J. Cell Biol. 217, 1303–1318. 10.1083/jcb.201709007 29463567 PMC5881506

[B173] Nikolic J. Civas A. Lama Z. Lagaudrière-Gesbert C. Blondel D. (2016). Rabies virus infection induces the formation of stress granules closely connected to the viral factories. PLoS Pathog. 12:e1005942. 10.1371/journal.ppat.1005942 27749929 PMC5066959

[B174] Nunes C. Mestre I. Marcelo A. Koppenol R. Matos C. A. Nóbrega C. (2019). MSGP: the first database of the protein components of the mammalian stress granules. Database 2019, 1–7. 10.1093/database/baz031 30820574 PMC6395795

[B175] Núñez R. D. Budt M. Saenger S. Paki K. Arnold U. Sadewasser A.. (2018). The RNA helicase DDX6 associates with RIG-I to augment induction of antiviral signaling. Int. J. Mol. Sci. 19, 1–14. 10.3390/ijms19071877 29949917 PMC6073104

[B176] Obrig T. Culp W. McKeehan W. Hardesty B. (1971). The mechanism by which cycloheximide and related glutarimide antibiotics inhibit peptidesynthesis on reticulocyte ribosomes. J. Biol. Chem. 246, 174–181. 5541758

[B177] Oh S.-W. Onomoto K. Wakimoto M. Onoguchi K. Ishidate F. Fujiwara T. (2016). Leader-containing uncapped viral transcript activates RIG-I in antiviral stress granules. PLoS Pathog. 12:e1005444 10.1371/journal.ppat.1005444 26862753 PMC4749238

[B178] Okonski K. M. Samuel C. E. (2012). Stress granule formation induced by measles virus is protein kinase PKR Dependent and impaired by RNA adenosine deaminase ADAR1. J. Virol. 87, 756–766. 10.1128/jvi.02270-12 23115276 PMC3554044

[B179] Onomoto K. Jogi M. Yoo J. S. Narita R. Morimoto S. Takemura A.. (2012). Critical role of an antiviral stress granule containing RIG-I and PKR in viral detection and innate immunity. PLoS ONE. 7:e43031. 10.1371/journal.pone.0043031 22912779 PMC3418241

[B180] Onomoto K. Yoneyama M. Fung G. Kato H. Fujita T. (2014). Antiviral innate immunity and stress granule responses. Trends Immunol. 35, 420–428. 10.1016/j.it.2014.07.006 25153707 PMC7185371

[B181] Oslowski C. M. Urano F. (2011). Measuring ER stress and the UPR using mammalian tissue culture system. Meth. Enzymol. 490, 71–92. 10.1016/B978-0-12-385114-7.00004-0 21266244 PMC3701721

[B182] Pager C. T. Schütz S. Abraham T. M. Luo G. Sarnow P. (2013). Modulation of hepatitis C virus RNA abundance and virus release by dispersion of processing bodies and enrichment of stress granules. Virology 435, 472–484. 10.1016/j.virol.2012.10.027 23141719 PMC3534916

[B183] Panas M. D. Ivanov P. Anderson P. (2016). Mechanistic insights into mammalian stress granule dynamics. J. Cell Biol. 215, 313–323. 10.1083/jcb.201609081 27821493 PMC5100297

[B184] Panas M. D. Schulte T. Thaa B. Sandalova T. Kedersha N. Achour A.. (2015). Viral and cellular proteins containing FGDF motifs bind G3BP to block stress granule formation. PLoS Pathog. 11:e1004659. 10.1371/journal.ppat.1004659 25658430 PMC4450067

[B185] Panas M. D. Varjak M. Lulla A. Er Eng K. Merits A. Karlsson Hedestam G. B.. (2012). Sequestration of G3BP coupled with efficient translation inhibits stress granules in Semliki Forest virus infection. Mol. Biol. Cell. 23, 4701–4712. 10.1091/mbc.e12-08-0619 23087212 PMC3521679

[B186] Pasieka T. J. Lu B. Crosby S. D. Wylie K. M. Morrison L. A. Alexander D. E.. (2008). Herpes simplex virus virion host shutoff attenuates establishment of the antiviral state. J. Virol. 82, 5527–5535. 10.1128/jvi.02047-07 18367525 PMC2395185

[B187] Patel J. McLeod L. E. Vries R. G. J. Flynn A. Wang X. Proud C. G. (2002). Cellular stresses profoundly inhibit protein synthesis and modulate the states of phosphorylation of multiple translation factors. Eur. J. Biochem. 269, 3076–3085. 10.1046/j.1432-1033.2002.02992.x 12071973

[B188] Pène V. Li Q. Sodroski C. Hsu C.-S. Liang T. J. (2015). Dynamic interaction of stress granules, DDX3X, and IKK-α Mediates multiple functions in hepatitis C virus infection. J. Virol. 89, 5462–5477. 10.1128/jvi.03197-14 25740981 PMC4442532

[B189] Pereira-Montecinos C. Valiente-Echeverría F. Soto-Rifo R. (2017). Epitranscriptomic regulation of viral replication. Biochim. Biophys. Acta 1860, 460–471. 10.1016/j.bbagrm.2017.02.002 28219769

[B190] Pérez-Vilar,ó G. Fernández-Carrillo C. Mensa L. Miquel R. Sanjuan X. Forns X. (2015). Hepatitis C virus infection inhibits P-body granule formation in human livers. J. Hepatol. 62, 785–790. 10.1016/j.jhep.2014.11.018 25463546

[B191] Pérez-Vilaró G. Scheller N. Saludes V. Díez J. (2012). Hepatitis C virus infection alters P-body composition but is independent of P-body granules. J. Virol. 86, 8740–8749. 10.1128/jvi.07167-11 22674998 PMC3421735

[B192] Pfaller C. K. Radeke M. J. Cattaneo R. Samuel C. E. (2013). Measles virus C protein impairs production of defective copyback double-stranded viral RNA and activation of protein kinase R. J. Virol. 88, 456–468. 10.1128/jvi.02572-13 24155404 PMC3911759

[B193] Phalora P. K. Sherer N. M. Wolinsky S. M. Swanson C. M. Malim M. H. (2012). HIV-1 replication and apobec3 antiviral activity are not regulated by P bodies. J. Virol. 86, 11712–11724. 10.1128/JVI.00595-12 22915799 PMC3486339

[B194] Pham A. M. Santa Maria F. G. Lahiri T. Friedman E. Mari,é I. J. Levy D. E. (2016). PKR transduces MDA5-dependent signals for type I IFN induction. PLoS Pathog. 12:e1005489. 10.1371/journal.ppat.1005489 26939124 PMC4777437

[B195] Pijlman G. P. Funk A. Kondratieva N. Leung J. Torres S. van der Aa L.. (2008). A highly structured, nuclease-resistant, noncoding RNA produced by flaviviruses is required for pathogenicity. Cell Host Microbe 4, 579–591. 10.1016/j.chom.2008.10.007 19064258

[B196] Piotrowska J. Hansen S. J. Park N. Jamka K. Sarnow P. Gustin K. E. (2010). Stable formation of compositionally unique stress granules in virus-infected cells. J. Virol. 84, 3654–3665. 10.1128/jvi.01320-09 20106928 PMC2838110

[B197] Poblete-Durán N. Prades-Pérez Y. Vera-Otarola J. Soto-Rifo R. Valiente-Echeverría F. (2016). Who regulates whom? An overview of RNA granules and viral infections. Viruses 8, 1–28. 10.3390/v8070180 27367717 PMC4974515

[B198] Qin Q. Carroll K. Hastings C. Miller C. L. (2011). Mammalian orthoreovirus escape from host translational shutoff correlates with stress granule disruption and is independent of eIF2 phosphorylation and PKR. J. Virol. 85, 8798–8810. 10.1128/jvi.01831-10 21715487 PMC3165827

[B199] Qin Q. Hastings C. Miller C. L. (2009). Mammalian orthoreovirus particles induce and are recruited into stress granules at early times postinfection. J. Virol. 83, 11090–11101. 10.1128/jvi.01239-09 19710141 PMC2772771

[B200] Raaben M. Groot Koerkamp M. J. A. Rottier P. J. M. de Haan C. A. M. (2007). Mouse hepatitis coronavirus replication induces host translational shutoff and mRNA decay, with concomitant formation of stress granules and processing bodies. Cell. Microbiol. 9, 2218–2229. 10.1111/j.1462-5822.2007.00951.x 17490409 PMC7162177

[B201] Rabouw H. H. Langereis M. A. Knaap R. C. M. Dalebout T. J. Canton J. Sola I.. (2016). Middle east respiratory coronavirus accessory protein 4a inhibits PKR-mediated antiviral stress responses. PLoS Pathog. 12:e1005982. 10.1371/journal.ppat.1005982 27783669 PMC5081173

[B202] Rai D. K. Lawrence P. Kloc A. Schafer E. Rieder E. (2015). Analysis of the interaction between host factor Sam68 and viral elements during foot-and-mouth disease virus infections. Virol. J. 12, 1–17. 10.1186/s12985-015-0452-8 26695943 PMC4689063

[B203] Raman S. N. T. Liu G. Pyo H. M. Cui Y. C. Xu F. Ayalew L. E. (2016). DDX3 interacts with influenza A virus NS1 and NP proteins and exerts antiviral function through regulation of stress granule formation. J. Virol. 90, 3661–3675. 10.1128/JVI.03010-15 26792746 PMC4794679

[B204] Ramaswami M. Taylor J. P. Parker R. (2013). Altered “Ribostasis”: RNA-protein granule formation or persistence in the development of degenerative disorders. Cell. 154, 727–736. . 10.1016/j.cell.2013.07.038 23953108 PMC3811119

[B205] Rao S. Amorim R. Niu M. Breton Y. Tremblay M. J. Mouland A. J. (2019a). Host mRNA decay proteins influence HIV-1 replication and viral gene expression in primary monocyte-derived macrophages. Retrovirology 16, 1–15. 10.1186/s12977-019-0465-2 30732620 PMC6367771

[B206] Rao S. Cinti A. Temzi A. Amorim R. You J. C. Mouland A. J. (2017). HIV-1 NC-induced stress granule assembly and translation arrest are inhibited by the dsRNA binding protein Staufen1. RNA 24, 219–236. 10.1261/rna.064618.117 29127210 PMC5769749

[B207] Rao S. Hassine S. Monette A. Amorim R. DesGroseillers L. Mouland A. (2019b). HIV-1 requires Staufen1 to dissociate stress granules and to produce infectious viral particles. RNA 25, 727–736. 10.1261/rna.069351.118 30902835 PMC6521601

[B208] Rayman J. B. Karl K. A. Kandel E. R. (2018). TIA-1 Self-Multimerization, phase separation, and recruitment into stress granules are dynamically regulated by Zn 2+. Cell Rep. 22, 59–71. 10.1016/j.celrep.2017.12.036 29298433

[B209] Reed J. C. Molter B. Geary C. D. McNevin J. McElrath J. Giri S.. (2012). HIV-1 Gag co-opts a cellular complex containing DDX6, a helicase that facilitates capsid assembly. J. Cell Biol. 198, 439–456. 10.1083/jcb.201111012 22851315 PMC3413349

[B210] Reed J. C. Westergreen N. Barajas B. C. Ressler D. T. B. Phuong D. J. Swain J. V.. (2018). Formation of RNA granule-derived capsid assembly intermediates appears to be conserved between human immunodeficiency virus type 1 and the nonprimate lentivirus feline immunodeficiency virus. J. Virol. 92, 1–30. 10.1128/jvi.01761-17 29467316 PMC5899207

[B211] Reineke L. C. Kedersha N. Langereis M. A. van Kuppeveld F. J. M. Lloyd R. E. (2015). Stress granules regulate double-stranded RNA-dependent protein kinase activation through a complex containing G3BP1 and caprin1. MBio 6, 1–12. 10.1128/mbio.02486-14 25784705 PMC4453520

[B212] Remenyi R. Zothner C. Merits A. Gao Y. Peckham M. Curd A.. (2018). Persistent replication of a chikungunya virus replicon in human cells is associated with presence of stable cytoplasmic granules containing nonstructural protein 3. J. Virol. 92, 1–24. 10.1128/jvi.00477-18 29875241 PMC6069192

[B213] Rhim J. S. Jordan L. E. Donald Mayor H. (1962). Cytochemical, fluorescent-antibody and electron microscopic studies on the growth of reovirus (ECHO 10) in tissue culture. Virology 355, 342–355. 10.1016/0042-6822(62)90125-3 14491769

[B214] Rojas M. Arias C. F. López S. (2010). Protein kinase R is responsible for the phosphorylation of eIF2α in rotavirus infection. J. Virol. 84, 10457–10466. 10.1128/jvi.00625-10 20631127 PMC2950594

[B215] Royall E. Doyle N. Abdul-Wahab A. Emmott E. Morley S. J. Goodfellow I.. (2015). Murine norovirus 1 (MNV1) replication induces translational control of the host by regulating eIF4E activity during infection. J. Biol. Chem. 290, 4748–4758. 10.1074/jbc.M114.602649 25561727 PMC4335213

[B216] Rozelle D. K. Filone C. M. Kedersha N. Connor J. H. (2014). Activation of stress response pathways promotes formation of antiviral granules and restricts virus replication. Mol. Cell. Biol. 34, 2003–2016. 10.1128/mcb.01630-13 24662051 PMC4019067

[B217] Ruggieri A. Dazert E. Metz P. Hofmann S. Bergeest J. P. Mazur J.. (2012). Dynamic oscillation of translation and stress granule formation mark the cellular response to virus infection. Cell Host Microbe 12, 71–85. 10.1016/j.chom.2012.05.013 22817989 PMC3873964

[B218] Scheller N. Mina L. B. Galao R. P. Chari A. Gimenez-Barcons M. Noueiry A.. (2009). Translation and replication of hepatitis C virus genomic RNA depends on ancient cellular proteins that control mRNA fates. Proc. Natl.Acad. Sci. U.S.A. 106, 13517–13522. 10.1073/pnas.0906413106 19628699 PMC2714764

[B219] Scholte F. E. M. Tas A. Albulescu I. C. Žusinaite E. Merits A. Snijder E.J.. (2015). Stress granule components G3BP1 and G3BP2 play a proviral role early in chikungunya virus replication. J. Virol. 89, 4457–4469. 10.1128/jvi.03612-14 25653451 PMC4442398

[B220] Schumann M. Gantke T. Muhlberger E. (2009). Ebola virus VP35 antagonizes PKR activity through its C-terminal interferon inhibitory domain. J. Virol. 83, 8993–8997. 10.1128/jvi.00523-09 19515768 PMC2738155

[B221] Schuster B. S. Reed E. H. Parthasarathy R. Jahnke C. N. Caldwell R. M. Bermudez J. G.. (2018). Controllable protein phase separation and modular recruitment to form responsive membraneless organelles. Nat. Commun. 9:2985. 10.1038/s41467-018-05403-1 30061688 PMC6065366

[B222] Sciortino M. T. Parisi T. Siracusano G. Mastino A. Taddeo B. Roizman B. (2013). The virion host shutoff RNase plays a key role in blocking the activation of protein kinase R in cells infected with herpes simplex virus 1. J. Virol. 87, 3271–3276. 10.1128/jvi.03049-12 23302873 PMC3592158

[B223] Senkevich T. G. Katsafanas G. C. Weisberg A. Olano L. R. Moss B. (2017). Identification of vaccinia virus replisome and transcriptome proteins by isolation of proteins on nascent DNA coupled with mass spectrometry. J. Virol. 91, 1–20. 10.1128/jvi.01015-17 28747503 PMC5599757

[B224] Seto E. Inoue T. Nakatani Y. Yamada M. Isomura H. (2014). Processing bodies accumulate in human cytomegalovirus-infected cells and do not affect viral replication at high multiplicity of infection. Virology 458–459, 151–61. 10.1016/j.virol.2014.04.022 24928047

[B225] Sharma N. R. Majerciak V. Kruhlak M. J. Zheng Z. M. (2017). KSHV inhibits stress granule formation by viral ORF57 blocking PKR activation. PLoS Pathog. 13:e1006677. 10.1371/journal.ppat.1006677 29084250 PMC5679657

[B226] Sharp P. A. (2009). The centrality of RNA. Cell 136, 577–580. 10.1016/j.cell.2009.02.007 19239877

[B227] Sidrauski C. McGeachy A. M. Ingolia N. T. Walter P. (2015). The small molecule ISRIB reverses the effects of eIF2α phosphorylation on translation and stress granule assembly. Elife 2015, 1–16. 10.7554/eLife.05033 PMC4341466 25719440

[B228] Silva P. A. G. C. Pereira C. F. Dalebout T. J. Spaan W. J. M. Bredenbeek P. J. (2010). An RNA pseudoknot is required for production of yellow fever virus subgenomic RNA by the host nuclease XRN1. J. Virol. 84, 11395–11406. 10.1128/jvi.01047-10 20739539 PMC2953177

[B229] Simpson-Holley M. Kedersha N. Dower K. Rubins K. H. Anderson P. Hensley L. E.. (2010). Formation of antiviral cytoplasmic granules during orthopoxvirus infection. J. Virol. 85, 1581–1593. 10.1128/jvi.02247-10 21147913 PMC3028896

[B230] Sivan G. Glushakow-Smith S. G. Katsafanas G. C. Americo J. L. Moss B. (2018). Human host range restriction of the vaccinia virus C7/K1 double deletion mutant is mediated by an atypical mode of translation inhibition. J. Virol. 92:e01329-18. 10.1128/jvi.01329-18 30209174 PMC6232495

[B231] Smith T. J. Ackland-Berglund C. E. Leib D. A. (2000). Herpes simplex virus virion host shutoff (vhs) activity alters periocular disease in mice. J. Virol. 74, 3598–3604. 10.1128/jvi.74.8.3598-3604.2000 10729135 PMC111869

[B232] Sokoloski K. J. Dickson A. M. Chaskey E. L. Garneau N. L. Wilusz C. J. Wilusz J. (2010). Sindbis virus usurps the cellular HuR protein to stabilize its transcripts and promote productive infections in mammalian and mosquito cells. Cell Host Microbe 8, 196–207. 10.1016/j.chom.2010.07.003 20709296 PMC2929003

[B233] Sola I. Galan C. Mateos-Gomez P. A. Palacio L. Zuniga S. Cruz J. L.. (2011). The Polypyrimidine tract-binding protein affects coronavirus RNA accumulation levels and relocalizes viral RNAs to novel cytoplasmic domains different from replication-transcription sites. J. Virol. 85, 5136–5149. 10.1128/jvi.00195-11 21411518 PMC3126201

[B234] Somasekharan S. P. El-Naggar A. Leprivier G. Cheng H. Hajee S. Grunewald T. G. P.. (2015). YB-1 regulates stress granule formation and tumor progression by translationally activating G3BP1. J. Cell Biol. 208, 913–929. 10.1083/jcb.201411047 25800057 PMC4384734

[B235] Soto-Rifo R. Valiente-Echeverria F. Rubilar P. S. Garcia-de-Gracia F. Ricci E. P. Limousin T.. (2014). HIV-2 genomic RNA accumulates in stress granules in the absence of active translation. Nucleic Acids Res. 42, 12861–12875. 10.1093/nar/gku1017 25352557 PMC4227750

[B236] Standart N. Weil D. (2018). P-Bodies: cytosolic droplets for coordinated mRNA storage. Trends Genet. 34, 612–626. 10.1016/j.tig.2018.05.005 29908710

[B237] Sun Y. Dong L. Yu S. Wang X. Zheng A. Zhang P.. (2017). Newcastle disease virus induces stable formation of bona fide stress granules to facilitate viral replication through manipulating host protein translation. FASEB J. 31, 1337–1353. 10.1096/fj.201600980R 28011649

[B238] Takahashi M. Higuchi M. Makokha G. N. Matsuki H. Yoshita M. Tanaka Y.. (2013). HTLV-1 Tax oncoprotein stimulates ROS production and apoptosis in T cells by interacting with USP10. Blood 122, 715–725. 10.1182/blood-2013-03-493718 23775713

[B239] Thedieck K. Holzwarth B. Prentzell M. T. Boehlke C. Kläsener K. Ruf S.. (2013). Inhibition of mTORC1 by astrin and stress granules prevents apoptosis in cancer cells. Cell 154, 859–874. 10.1016/j.cell.2013.07.031 23953116

[B240] Thomas M. G. Martinez Tosar L. J. Desbats M. A. Leishman C. C. Boccaccio G. L. (2009). Mammalian Staufen 1 is recruited to stress granules and impairs their assembly. J. Cell Sci. 122, 563–573. 10.1242/jcs.038208 19193871 PMC2714435

[B241] Toro-Ascuy D. Rojas-Araya B. Valiente-Echeverría F. Soto-Rifo R. (2016). Interactions between the HIV-1 unspliced mRNA and host mRNA decay machineries. Viruses. 8:E320. 10.3390/v8110320 27886048 PMC5127034

[B242] Toth A. M. Li Z. Cattaneo R. Samuel C. E. (2009). RNA-specific adenosine deaminase ADAR1 suppresses measles virus-induced apoptosis and activation of protein kinase PKR. J. Biol. Chem. 284, 29350–29356. 10.1074/jbc.M109.045146 19710021 PMC2785566

[B243] Tourrière H. Chebli K. Zekri L. Courselaud B. Blanchard J. M. Bertrand E.. (2003). The RasGAP-associated endoribonuclease G3BP assembles stress granules. J. Cell Biol. 160, 823–831. 10.1083/jcb.200212128 12642610 PMC2173781

[B244] Trifinopoulos J. Nguyen L.-T. von Haeseler A. Minh B. Q. (2016). W-IQ-TREE: a fast online phylogenetic tool for maximum likelihood analysis. Nucleic Acids Res. 44, W232–W235. 10.1093/nar/gkw256 27084950 PMC4987875

[B245] Valiente-Echeverría F. Hermoso M. A. Soto-Rifo R. (2015). RNA helicase DDX3: at the crossroad of viral replication and antiviral immunity. Rev. Med. Virol. 25, 286–299. 10.1002/rmv.1845 26174373

[B246] Valiente-Echeverría F. Melnychuk L. Vyboh K. Ajamian L. Gallouzi I. E. Bernard N.. (2014). EEF2 and Ras-GAP SH3 domain-binding protein (G3BP1) modulate stress granule assembly during HIV-1 infection. Nat. Commun. 5:4819. 10.1038/ncomms5819 25229650 PMC4978539

[B247] Venticinque L. Meruelo D. (2010). Sindbis viral vector induced apoptosis requires translational inhibition and signaling through Mcl-1 and Bak. Mol. Cancer. 9, 1–16. 10.1186/1476-4598-9-37 20152035 PMC2843653

[B248] Visser L. J. Medina G. N. Rabouw H. H. Groot R. J. Langereis M. A. de los Santos T. (2019). Foot-and-mouth disease virus leader protease cleaves G3BP1 and G3BP2 and inhibits stress granule formation linda. J. Virol. 93, 1–16. 10.1128/JVI.00922-18 PMC6321903 30404792

[B249] Walsh D. Arias C. Perez C. Halladin D. Escandon M. Ueda T.. (2008). Eukaryotic translation initiation factor 4F architectural alterations accompany translation initiation factor redistribution in poxvirus-infected cells. Mol. Cell. Biol. 28, 2648–2658. 10.1128/mcb.01631-07 18250159 PMC2293122

[B250] Wang H. Chang L. Wang X. Su A. Feng C. Fu Y.. (2016). MOV10 interacts with enterovirus 71 genomic 5′UTR and modulates viral replication. Biochem. Biophys. Res. Commun. 479, 571–577. 10.1016/j.bbrc.2016.09.112 27666477

[B251] Wang P. Song W. Mok B. W.-Y. Zhao P. Qin K. Lai A.. (2009). Nuclear factor 90 negatively regulates influenza virus replication by interacting with viral nucleoprotein. J. Virol. 83, 7850–7861. 10.1128/jvi.00735-09 19494010 PMC2715781

[B252] Wang W.-T. Tsai T.-Y. Chao C.-H. Lai B.-Y. Wu Lee Y.-H. (2015). Y-box binding protein 1 stabilizes hepatitis C virus NS5A via phosphorylation-mediated interaction with NS5A to regulate viral propagation. J. Virol. 89, 11584–11602. 10.1128/jvi.01513-15 26355086 PMC4645663

[B253] Wang X. Liao Y. Yap P. L. Png K. J. Tam J. P. Liu D. X. (2009). Inhibition of protein kinase R activation and upregulation of GADD34 expression play a synergistic role in facilitating coronavirus replication by maintaining *de novo* protein synthesis in virus-infected cells. J. Virol. 83, 12462–12472. 10.1128/jvi.01546-09 19776135 PMC2786722

[B254] Wang Z. Mir M. A. (2014). Andes virus nucleocapsid protein interrupts protein kinase R dimerization to counteract host interference in viral protein synthesis. J. Virol. 89, 1628–1639. 10.1128/jvi.02347-14 25410857 PMC4300761

[B255] Ward A. M. Bidet K. Yinglin A. Ler S. G. Hogue K. Blackstock W.. (2011). Quantitative mass spectrometry of DENV-2 RNA-interacting proteins reveals that the DEAD-box RNA helicase DDX6 binds the DB1 and DB2 3′ UTR structures. RNA Biol. 8, 1173–86. 10.4161/rna.8.6 21957497 PMC3256426

[B256] Weissbach R. Scadden A. D. J. (2012). Tudor-SN and ADAR1 are components of cytoplasmic stress granules. RNA 18, 462–471. 10.1261/rna.027656.111 22240577 PMC3285934

[B257] Wen X. Huang X. Mok B. W.-Y. Chen Y. Zheng M. Wang P.. (2014). NF90 exerts antiviral activity through regulation of PKR phosphorylation and stress granules in infected cells. J. Immunol. 192, 3753–3764. 10.4049/jimmunol.1302813 24623135

[B258] Wheeler J. R. Matheny T. Jain S. Abrisch R. Parker R. (2016). Distinct stages in stress granule assembly and disassembly. Elife 5, 1–25. 10.7554/elife.18413 27602576 PMC5014549

[B259] White J. P. Cardenas A. M. Marissen W. E. Lloyd R. E. (2007). Inhibition of cytoplasmic mRNA stress granule formation by a viral proteinase. Cell Host Microbe 2, 295–305. 10.1016/j.chom.2007.08.006 18005751

[B260] White J. P. Lloyd R. E. (2011). Poliovirus unlinks TIA1 aggregation and mRNA stress granule formation. J. Virol. 85, 12442–12454. 10.1128/jvi.05888-11 21957303 PMC3209409

[B261] Wilbertz J. H. Voigt F. Horvathova I. Roth G. Zhan Y. Chao J. A. (2018). Single-molecule imaging of mRNA localization and regulation during the integrated stress response. Mol. Cell 73, 946–958. 10.1016/j.molcel.2018.12.006 30661979

[B262] Williams B. R. G. (2001). Signal integration via PKR. Sci. Signal. 2001:re2. 10.1126/stke.2001.89.re2 11752661

[B263] Wong J. Si X. Angeles A. Zhang J. Shi J. Fung G.. (2013). Cytoplasmic redistribution and cleavage of AUF1 during coxsackievirus infection enhance the stability of its viral genome. FASEB J. 27, 2777–2787. 10.1096/fj.12-226498 23572232

[B264] Wu S. Wang Y. Lin L. Si X. Wang T. Zhong X.. (2014). Protease 2A induces stress granule formation during coxsackievirus B3 and enterovirus 71 infections. Virol. J. 11, 1–10. 10.1186/s12985-014-0192-1 25410318 PMC4247557

[B265] Xue M. Fu F. Ma Y. Zhang X. Li L. Feng L.. (2018). The PERK arm of the unfolded protein response negatively regulates transmissible gastroenteritis virus replication by suppressing protein translation and promoting type I interferon production. J. Virol. 92, 1–21. 29769338 10.1128/JVI.00431-18 PMC6052291

[B266] Yang X. Hu Z. Fan S. Zhang Q. Zhong Y. Guo D.. (2018b). Picornavirus 2A protease regulates stress granule formation to facilitate viral translation. PLoS Pathog. 14:e1006901. 10.1371/journal.ppat.1006901 29415027 PMC5819834

[B267] Yang X. Hu Z. Zhang Q. Fan S. Zhong Y. Guo D.. (2018a). SG formation relies on eIF4GI-G3BP interaction which is targeted by picornavirus stress antagonists. Cell Discov. 5, 1–14. 10.1038/s41421-018-0068-4 30603102 PMC6312541

[B268] Ye X. Pan T. Wang D. Fang L. Ma J. Zhu X.. (2018). Foot-and-mouth disease virus counteracts on internal ribosome entry site suppression by G3BP1 and inhibits G3BP1-mediated stress granule assembly via post-translational mechanisms. Front. Immunol. 9:1142. 10.3389/fimmu.2018.01142 29887867 PMC5980976

[B269] Ye Y. Hauns K. Langland J. O. Jacobs B. L. Hogue B. G. (2007). Mouse hepatitis coronavirus A59 nucleocapsid protein is a type I interferon antagonist. J. Virol. 81, 2554–2563. 10.1128/JVI.01634-06 17182678 PMC1865977

[B270] Yoshida A. Kawabata R. Honda T. Tomonaga K. Sakaguchi T. Irie T. (2015). IFN-ß-inducing, unusual viral RNA species produced by paramyxovirus infection accumulated into distinct cytoplasmic structures in an RNA-type-dependent manner. Front. Microbiol. 6:804 10.3389/fmicb.2015.00804 26300870 PMC4523817

[B271] Zaborowska I. Kellner K. Henry M. Meleady P. Walsh D. (2012). Recruitment of host translation initiation factor eIF4G by the vaccinia virus ssDNA-binding protein I3. Virology 425, 11–22. 10.1016/j.virol.2011.12.022 22280895

[B272] Zhai X. Wu S. Lin L. Wang T. Zhong X. Chen Y.. (2018). Stress granule formation is one of the early antiviral mechanisms for host cells against coxsackievirus B infection. Virol. Sin. 33, 314–322. 10.1007/s12250-018-0040-3 29959686 PMC6178097

[B273] Zhang H. Chen N. Li P. Pan Z. Ding Y. Zou D.. (2016). The nuclear protein Sam68 is recruited to the cytoplasmic stress granules during enterovirus 71 infection. Microb. Pathog. 96, 58–66. 10.1016/j.micpath.2016.04.001 27057671

[B274] Zhang Y. Yao L. Xu X. Han H. Li P. Zou D.. (2018). Enterovirus 71 inhibits cytoplasmic stress granule formation during the late stage of infection. Virus Res. 255, 55–67. 10.1016/j.virusres.2018.07.006 30006004

[B275] Zhou Y. Fang L. Wang D. Cai K. Chen H. Xiao S. (2017). Porcine reproductive and respiratory syndrome virus infection induces stress granule formation depending on protein kinase R-like endoplasmic reticulum kinase (PERK) in MARC-145 Cells. Front. Cell. Infect. Microbiol. 7:111. 10.3389/fcimb.2017.00111 28421170 PMC5378712

[B276] Zhu Y. Wang B. Huang H. Zhao Z. (2016). Enterovirus 71 induces anti-viral stress granule-like structures in RD cells. Biochem. Biophys. Res. Commun. 476, 212–217. 10.1016/j.bbrc.2016.05.094 27216457 PMC7124267

[B277] Ziehr B. Vincent H. A. Moorman N. J. (2016). Human cytomegalovirus pTRS1 and pIRS1 antagonize protein kinase R to facilitate virus replication. J. Virol. 90, 3839–3848. 10.1128/jvi.02714-15 26819306 PMC4810536

